# Supplementary material for: Integrating multimodal and multiscale connectivity blueprints of the human cerebral cortex in health and disease
Source: PLoS Biol. 2023 Sep 25;21(9):e3002314. doi: 10.1371/journal.pbio.3002314 (PMC10553842; doi:10.1371/journal.pbio.3002314)
Supplement: S1 Fig — (PDF) [file pbio.3002314.s001.pdf]

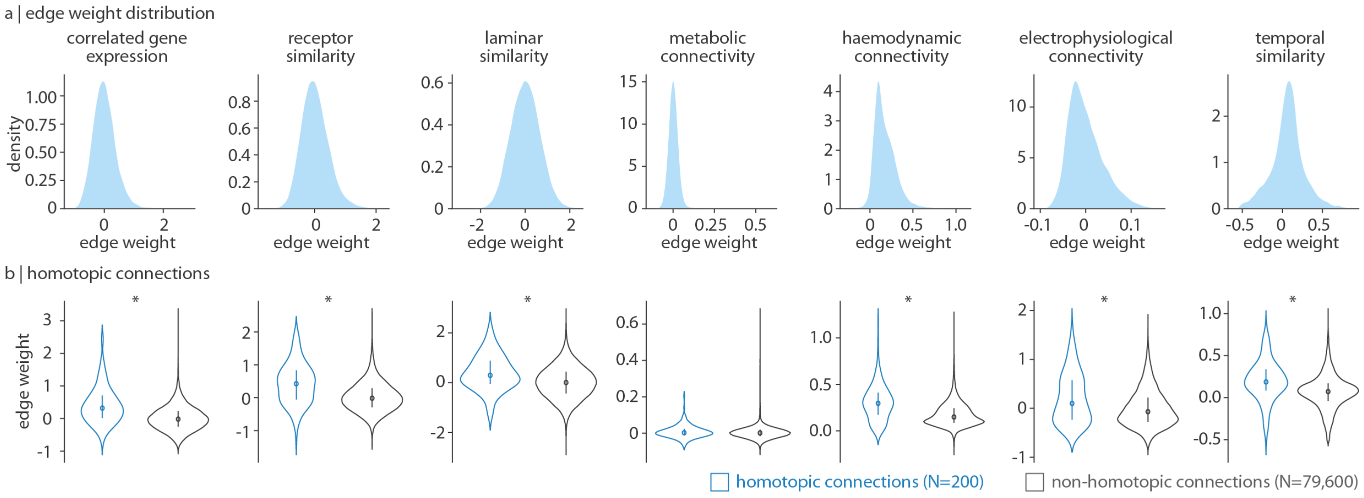

Figure S1. **Distributions of normalized edge weight for each connectivity mode |** (a) Edge weight distributions of each normalized connectivity mode. (b) Edge weight distributions for homotopic ($N = 200$ edges; blue) and non-homotopic ($N = 79\,600$; grey) edges. Significance was determined using two-tailed Welch's t-test. The data underlying this figure can be found at https://github.com/netneurolab/hansen_many_networks.
