# Supplementary material for: Integrating multimodal and multiscale connectivity blueprints of the human cerebral cortex in health and disease
Source: PLoS Biol. 2023 Sep 25;21(9):e3002314. doi: 10.1371/journal.pbio.3002314 (PMC10553842; doi:10.1371/journal.pbio.3002314)
Supplement: S2 Fig — (PDF) [file pbio.3002314.s002.pdf]

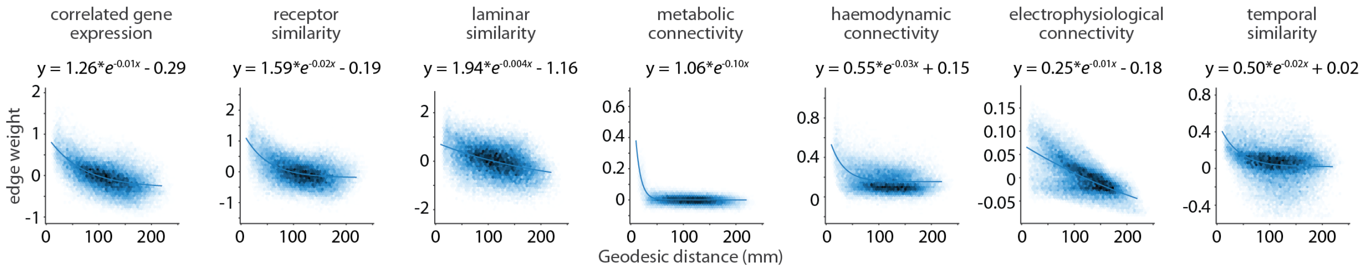

Figure S2. **Relationship between edge strength and geodesic distance |** Edge weights between pairs of cortical regions within the same hemisphere decrease with geodesic distance across all seven connectivity modes. Darker colours represent a greater density of points. This relationship is better fit with an exponential rather than linear function (exponential equations shown on top). Note that geodesic distance—the distance along the surface of the cortex between two regions—is only computed within hemispheres so between-hemisphere edges are excluded from the analysis. The data underlying this figure can be found at https://github.com/netneurolab/hansen_many_networks.
