# Supplementary material for: Integrating multimodal and multiscale connectivity blueprints of the human cerebral cortex in health and disease
Source: PLoS Biol. 2023 Sep 25;21(9):e3002314. doi: 10.1371/journal.pbio.3002314 (PMC10553842; doi:10.1371/journal.pbio.3002314)
Supplement: S3 Fig — (PDF) [file pbio.3002314.s003.pdf]

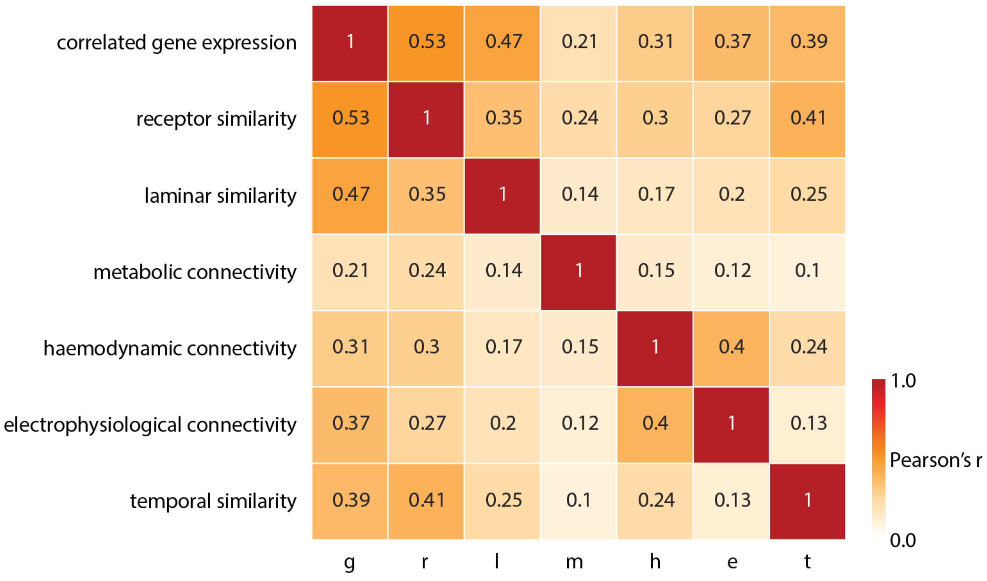


Figure S3. **Edge-wise correspondence between connectivity modes |** Pearson's correlation of the upper triangle of every pair of connectivity modes included in the analyses. The data underlying this figure can be found at https://github.com/netneurolab/hansen_many_networks.
