# Supplementary material for: Integrating multimodal and multiscale connectivity blueprints of the human cerebral cortex in health and disease
Source: PLoS Biol. 2023 Sep 25;21(9):e3002314. doi: 10.1371/journal.pbio.3002314 (PMC10553842; doi:10.1371/journal.pbio.3002314)
Supplement: S4 Fig — (PDF) [file pbio.3002314.s004.pdf]

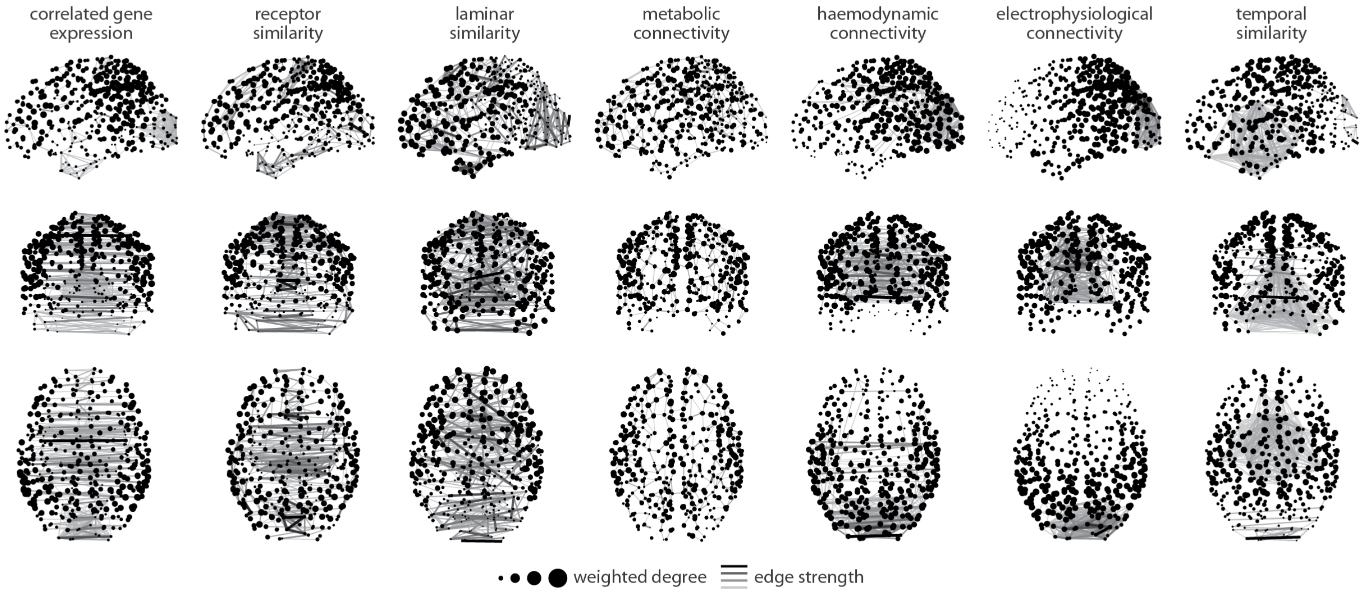

Figure S4. **Alternative views of the strongest edges in each connectivity mode |** For each connectivity mode, we plot the 0.5% strongest edges. Darker and thicker lines indicate stronger edges. Points represent cortical regions and are sized according to the sum of edge weights (weighted degree). Here we show sagittal, coronal, and axial views to complement Fig. 3a. The data underlying this figure can be found at https://github.com/netneurolab/hansen_many_networks.
