# Supplementary material for: Integrating multimodal and multiscale connectivity blueprints of the human cerebral cortex in health and disease
Source: PLoS Biol. 2023 Sep 25;21(9):e3002314. doi: 10.1371/journal.pbio.3002314 (PMC10553842; doi:10.1371/journal.pbio.3002314)
Supplement: S5 Fig — (PDF) [file pbio.3002314.s005.pdf]

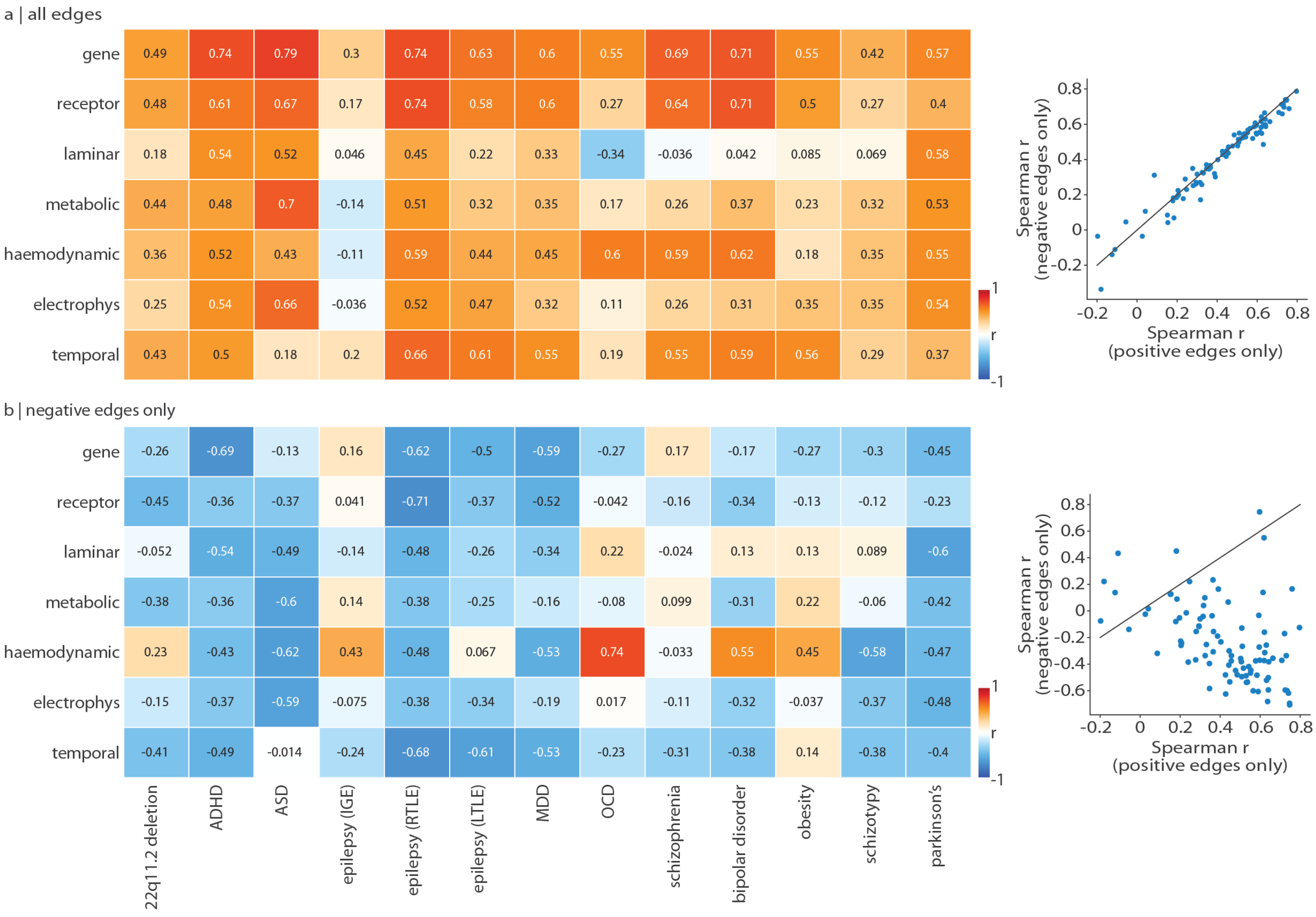

Figure S5. **Investigating the influence of negative edges in disease exposure |** We repeat the procedure in Fig. 4 but instead of retaining only positive edges, we retain (a) all edges, and (b) only negative edges. Scatter plots compare the original (positive edges only; x-axis) and updated (y-axis) Spearman's r correlation coefficients, with the identity line shown. The data underlying this figure can be found at https://github.com/netneurolab/hansen_many_networks.
