# Supplementary material for: Integrating multimodal and multiscale connectivity blueprints of the human cerebral cortex in health and disease
Source: PLoS Biol. 2023 Sep 25;21(9):e3002314. doi: 10.1371/journal.pbio.3002314 (PMC10553842; doi:10.1371/journal.pbio.3002314)
Supplement: S6 Fig — (PDF) [file pbio.3002314.s006.pdf]

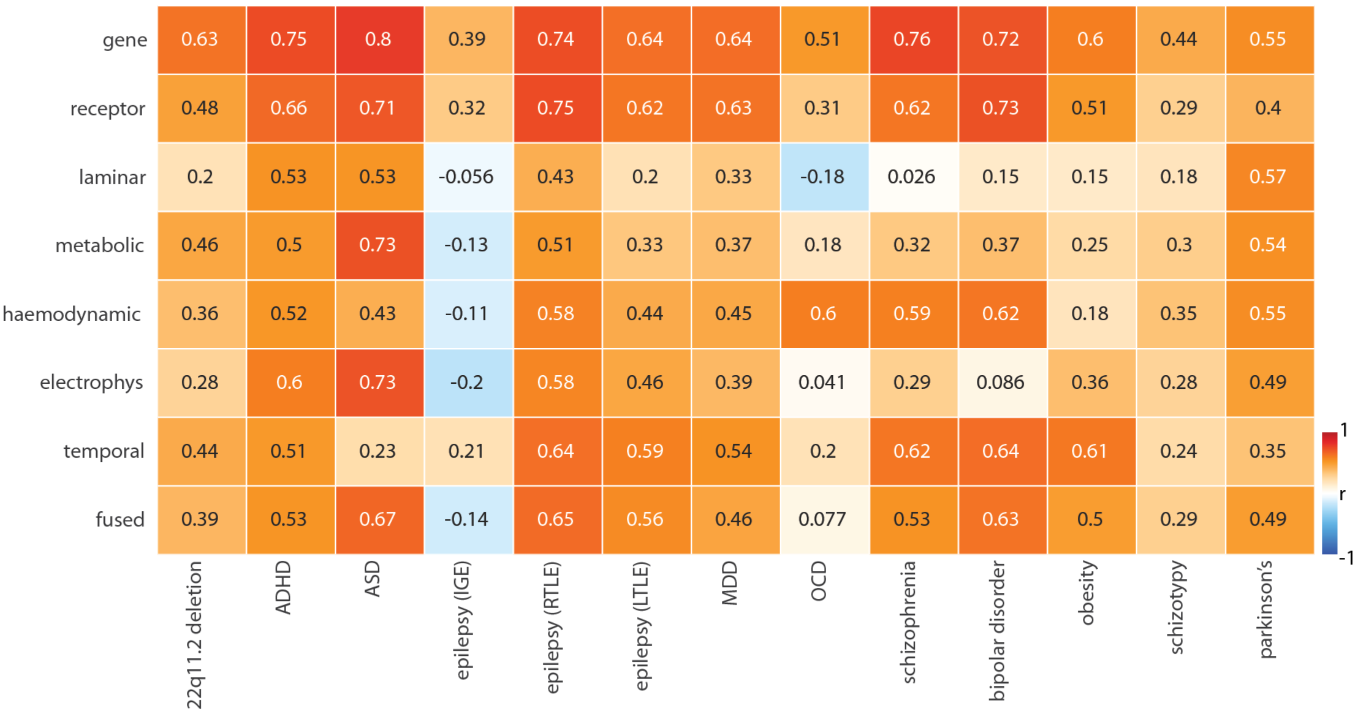

Figure S6. **Contributions of connectivity modes including the fused network to disease vulnerability |** We repeat the procedure in Fig. 4 using the fused network from the analysis in Fig. 6 (bottom row). The first seven rows of the heatmap, corresponding to the seven connectivity modes, are identical to those shown in Fig. 4 and are repeated to facilitate comparison with the fused network. We find that the fused network performs average, which may reflect that by fusing connectivity modes, the benefits of molecular connectivity modes in predicting disease patterns are washed out by the dynamic modes which perform less well. The data underlying this figure can be found at https://github.com/netneurolab/hansen_many_networks.
