# Supplementary material for: Integrating multimodal and multiscale connectivity blueprints of the human cerebral cortex in health and disease
Source: PLoS Biol. 2023 Sep 25;21(9):e3002314. doi: 10.1371/journal.pbio.3002314 (PMC10553842; doi:10.1371/journal.pbio.3002314)
Supplement: S7 Fig — (PDF) [file pbio.3002314.s007.pdf]

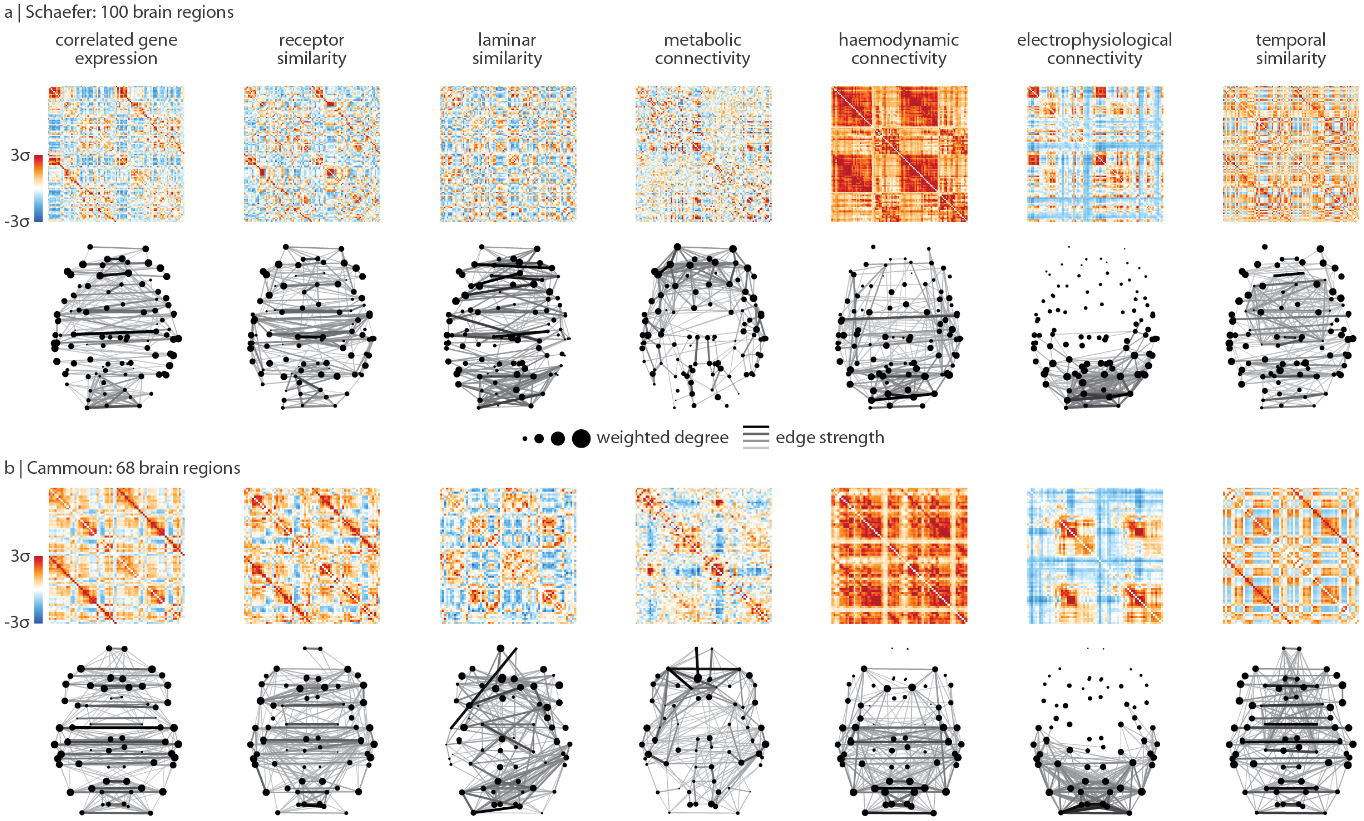

Figure S7. **Replication using alternative parcellations |** Analyses were repeated using (a) the 100-region Schaefer parcellation and (b) the 68-region Cammoun parcellation (regionally equivalent to the Desikan-Killiany atlas) [1–3]. We show each network as well as the the 5% (for Schaefer) or 10% (for Cammoun) strongest edges of the network. The data underlying this figure can be found at https://github.com/netneurolab/hansen_many_networks.

References

[1] Cammoun, L., Gigandet, X., Meskaldji, D., Thiran, J. P., Sporns, O., Do, K. Q., Maeder, P., Meuli, R., and Hagmann, P. (2012). Mapping the human connectome at multiple scales with diffusion spectrum mri. *J Neurosci Meth*, 203(2):386–397.

[2] Desikan, R. S., Ségonne, F., Fischl, B., Quinn, B. T., Dickerson, B. C., Blacker, D., Buckner, R. L., Dale, A. M., Maguire, R. P., Hyman, B. T., et al. (2006). An automated labeling system for subdividing the human cerebral cortex on mri scans into gyral based regions of interest. *NeuroImage*, 31(3):968–980.

[3] Schaefer, A., Kong, R., Gordon, E. M., Laumann, T. O., Zuo, X.-N., Holmes, A. J., Eickhoff, S. B., and Yeo, B. T. (2018). Local-global parcellation of the human cerebral cortex from intrinsic functional connectivity mri. *Cerebral cortex*, 28(9):3095–3114.
