# Supplementary material for: Integrating multimodal and multiscale connectivity blueprints of the human cerebral cortex in health and disease
Source: PLoS Biol. 2023 Sep 25;21(9):e3002314. doi: 10.1371/journal.pbio.3002314 (PMC10553842; doi:10.1371/journal.pbio.3002314)
Supplement: S8 Fig — (PDF) [file pbio.3002314.s008.pdf]

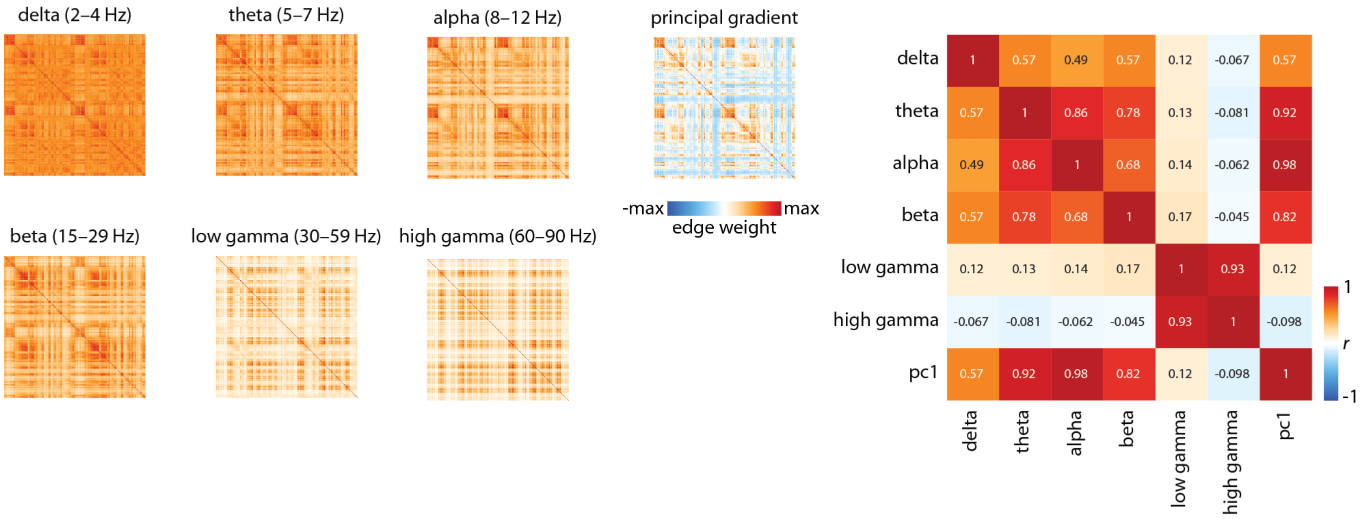

Figure S8. **MEG connectomes across frequency bands |** Amplitude envelope correlations were performed between time-series at each pair of cortical regions for six canonical frequency bands separately (delta (2–4 Hz), theta (5–7 Hz), alpha (8–12 Hz), beta (15–29 Hz), low gamma (30–59 Hz), high gamma (60–90 Hz). The electrophysiological connectivity mode used in the present analyses is the first principal component of the vectorized upper triangles of all six frequency-dependent connectivity matrices. On the right we show Pearson's correlations between the six frequency-dependent connectivity matrices and the principal gradient, showing that the first gradient is a good proxy for electrophysiological connectivity. The data underlying this figure can be found at https://github.com/netneurolab/hansen_many_networks.
