# Supplementary material for: Integrating multimodal and multiscale connectivity blueprints of the human cerebral cortex in health and disease
Source: PLoS Biol. 2023 Sep 25;21(9):e3002314. doi: 10.1371/journal.pbio.3002314 (PMC10553842; doi:10.1371/journal.pbio.3002314)
Supplement: S9 Fig — (PDF) [file pbio.3002314.s009.pdf]

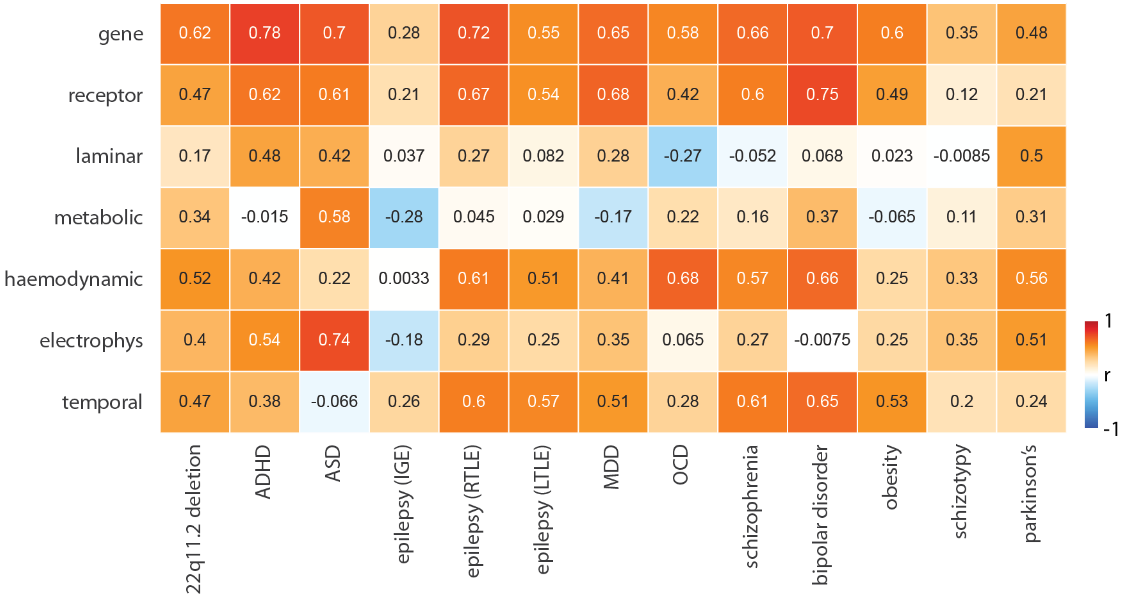

Figure S9. **Contributions of connectivity modes to disease vulnerability** | We repeat the procedure in Fig. 4 after regressing the exponential relationship with Euclidean distance (shown in Fig. 1b) from each connectivity mode. The data underlying this figure can be found at https://github.com/netneurolab/hansen_many_networks.
