# Supplementary material for: Integrating multimodal and multiscale connectivity blueprints of the human cerebral cortex in health and disease
Source: PLoS Biol. 2023 Sep 25;21(9):e3002314. doi: 10.1371/journal.pbio.3002314 (PMC10553842; doi:10.1371/journal.pbio.3002314)
Supplement: S1 Table — (PDF) [file pbio.3002314.s010.pdf]

Receptor/ transporter	Neurotransmitter	Tracer	Measure	N	References
D_1	dopamine	$[^{11}C]$SCH23390	BP_{ND}	13 (7)	[16]
D_2	dopamine	$[^{11}C]$FLB-457	BP_{ND}	92 (49)	[30, 31, 34, 36, 37]
DAT	dopamine	$[^{123}I]$-FP-CIT	SUVR	174 (65)	[10]
NET	norepinephrine	$[^{11}C]$MRB	BP_{ND}	77 (27)	[5, 7, 8, 29]
5-HT_{1A}	serotonin	$[^{11}C]$WAY-100635	BP_{ND}	35 (17)	[33]
5-HT_{1B}	serotonin	$[^{11}C]$P943	BP_{ND}	88 (24)	[3, 12, 18–20, 25, 32, 33]
5-HT_{2A}	serotonin	$[^{11}C]$Cimbi-36	B_{max}	29 (14)	[6]
5-HT_4	serotonin	$[^{11}C]$SB207145	B_{max}	59 (18)	[6]
5-HT_6	serotonin	$[^{11}C]$GSK215083	BP_{ND}	30 (0)	[26, 27]
5-HTT	serotonin	$[^{11}C]$DASB	B_{max}	100 (71)	[6]
$\alpha_4\beta_2$	acetylcholine	$[^{18}F]$flubatine	V_T	30 (10)	[2, 15]
M_1	acetylcholine	$[^{11}C]$LSN3172176	BP_{ND}	24 (11)	[21]
VAChT	acetylcholine	$[^{18}F]$FEOBV	SUVR	30 (18)	[1, 4, 14]
$mGluR_5$	glutamate	$[^{11}C]$ABP688	BP_{ND}	123 (71)	[9, 14, 35]
$GABA_{A/BZ}$	GABA	$[^{11}C]$flumazenil	B_{max}	16 (9)	[23]
H_3	histamine	$[^{11}C]$GSK189254	V_T	8 (1)	[13]
CB_1	cannabinoid	$[^{11}C]$OMAR	V_T	77 (28)	[11, 22, 24, 28]
MOR	opioid	$[^{11}C]$carfentanil	BP_{ND}	204 (72)	[17]

TABLE S1. **Neurotransmitter receptors and transporters included in receptor similarity** | BP_{ND} = non-displaceable binding potential; V_T = tracer distribution volume; B_{max} = density (pmol/ml) converted from binding potential (5-HT) or distributional volume (GABA) using autoradiography-derived densities; SUVR = standard uptake value ratio. Values in parentheses (under N) indicate number of females. This table is adapted from Table 1 of [14] which is licensed under a Creative Commons Attribution 4.0 International License (https://creativecommons.org/licenses/by/4.0/).

References

[1] Aghourian, M., Legault-Denis, C., Soucy, J., Rosa-Neto, P., Gauthier, S., Kostikov, A., Gravel, P., and Bedard, M. (2017). Quantification of brain cholinergic denervation in alzheimer's disease using pet imaging with [18 f]-feobv. *Molecular psychiatry*, 22(11):1531–1538.

[2] Baldassarri, S. R., Hillmer, A. T., Anderson, J. M., Jatlow, P., Nabulsi, N., Labaree, D., Cosgrove, K. P., O'Malley, S. S., Eissenberg, T., Krishnan-Sarin, S., et al. (2018). Use of electronic cigarettes leads to significant beta2-nicotinic acetylcholine receptor occupancy: evidence from a pet imaging study. *Nicotine and Tobacco Research*, 20(4):425–433.

[3] Baldassarri, S. R., Park, E., Finnema, S. J., Planeta, B., Nabulsi, N., Najafzadeh, S., Ropchan, J., Huang, Y., Hannestad, J., Maloney, K., et al. (2020). Inverse changes in raphe and cortical 5-ht1b receptor availability after acute tryptophan depletion in healthy human subjects. *Synapse*, 74(10):e22159.

[4] Bedard, M.-A., Aghourian, M., Legault-Denis, C., Postuma, R. B., Soucy, J.-P., Gagnon, J.-F., Pelletier, A., and Montplaisir, J. (2019). Brain cholinergic alterations in idiopathic rem sleep behaviour disorder: a pet imaging study with 18f-feobv. *Sleep medicine*, 58:35–41.

[5] Belfort-DeAguiar, R., Gallezot, J.-D., Hwang, J. J., Elshafie, A., Yeckel, C. W., Chan, O., Carson, R. E., Ding, Y.-S., and Sherwin, R. S. (2018). Noradrenergic activity in the human brain: a mechanism supporting the defense against hypoglycemia. *The Journal of Clinical Endocrinology & Metabolism*, 103(6):2244–2252.

[6] Beliveau, V., Ganz, M., Feng, L., Ozenne, B., Højgaard, L., Fisher, P. M., Svarer, C., Greve, D. N., and Knudsen, G. M. (2017). A high-resolution in vivo atlas of the human brain's serotonin system. *Journal of Neuroscience*, 37(1):120–128.

[7] Chiang-shan, R. L., Potenza, M. N., Lee, D. E., Planeta, B., Gallezot, J.-D., Labaree, D., Henry, S., Nabulsi, N., Sinha, R., Ding, Y.-S., et al. (2014). Decreased norepinephrine transporter availability in obesity: positron emission tomography imaging with (s, s)-[11c] o-methylreboxetine. *Neuroimage*, 86:306–310.

[8] Ding, Y.-S., Singhal, T., Planeta-Wilson, B., Gallezot, J.-D., Nabulsi, N., Labaree, D., Ropchan, J., Henry, S., Williams, W., Carson, R. E., et al. (2010). Pet imaging of the effects of age and cocaine on the norepinephrine transporter in the human brain using (s, s)-[11c] o-methylreboxetine and hrrt. *Synapse*, 64(1):30–38.

[9] DuBois, J. M., Rousset, O. G., Rowley, J., Porras-Betancourt, M., Reader, A. J., Labbe, A., Massarweh, G., Soucy, J.-P., Rosa-Neto, P., and Kobayashi, E. (2016). Characterization of age/sex and the regional distribution of mglur5 availability in the healthy human brain measured by high-resolution [11 c] abp688 pet. *European journal of nuclear medicine and molecular imaging*, 43(1):152–162.

[10] Dukart, J., Holiga, Š., Chatham, C., Hawkins, P., Forsyth, A., McMillan, R., Myers, J., Lingford-Hughes, A. R., Nutt, D. J., Merlo-Pich, E., et al. (2018). Cerebral blood flow predicts differential neurotransmitter activity. *Scientific reports*, 8(1):1–11.

[11] D'Souza, D. C., Cortes-Briones, J. A., Ranganathan, M., Thurnauer, H., Creatura, G., Surti, T., Planeta, B., Neumeister, A., Pittman, B., Normandin, M. D., et al. (2016). Rapid changes in cannabinoid 1 receptor availability in cannabis-dependent male subjects after abstinence from cannabis. *Biological psychiatry: cognitive neuroscience and neuroimaging*, 1(1):60–67.

[12] Gallezot, J.-D., Nabulsi, N., Neumeister, A., Planeta-Wilson, B., Williams, W. A., Singhal, T., Kim, S., Maguire, R. P., McCarthy, T., Frost, J. J., et al. (2010). Kinetic modeling of the serotonin 5-ht1b receptor radioligand [11c] p943 in humans. *Journal of Cerebral Blood Flow & Metabolism*, 30(1):196–210.

[13] Gallezot, J.-D., Planeta, B., Nabulsi, N., Palumbo, D., Li, X., Liu, J., Rowinski, C., Chidsey, K., Labaree, D., Ropchan, J., et al. (2017). Determination of receptor occupancy in the presence of mass dose:[11c] gsk189254 pet imaging of histamine h3 receptor occupancy by pf-03654746. *Journal of Cerebral Blood Flow & Metabolism*, 37(3):1095–1107.

[14] Hansen, J. Y., Shafiei, G., Markello, R. D., Smart, K., Cox, S. M., Nørgaard, M., Beliveau, V., Wu, Y., Gallezot, J.-D., Aumont, É., et al. (2022). Mapping neurotransmitter systems to the structural and functional organization of the human neocortex. *Nature Neuroscience*, pages 1–13.

[15] Hillmer, A. T., Esterlis, I., Gallezot, J.-D., Bois, F., Zheng, M.-Q., Nabulsi, N., Lin, S.-F., Papke, R., Huang, Y., Sabri, O., et al. (2016). Imaging of cerebral $\alpha4\beta2$* nicotinic acetylcholine receptors with (-)-[18f] flubatine pet: Implementation of bolus plus constant infusion and sensitivity to acetylcholine in human brain. *Neuroimage*, 141:71–80.

[16] Kaller, S., Rullmann, M., Patt, M., Becker, G.-A., Luthardt, J., Girbardt, J., Meyer, P. M., Werner, P., Barthel, H., Bresch, A., et al. (2017). Test–retest measurements of dopamine d 1-type receptors using simultaneous pet/mri imaging. *European journal of nuclear medicine and molecular imaging*, 44(6):1025–1032.

[17] Kantonen, T., Karjalainen, T., Isojärvi, J., Nuutila, P., Tuisku, J., Rinne, J., Hietala, J., Kaasinen, V., Kalliokoski, K., Scheinin, H., et al. (2020). Interindividual variability and lateralization of μ-opioid receptors in the human brain. *NeuroImage*, 217:116922.

[18] Matuskey, D., Bhagwagar, Z., Planeta, B., Pittman, B., Gallezot, J.-D., Chen, J., Wanyiri, J., Najafzadeh, S., Ropchan, J., Geha, P., et al. (2014). Reductions in brain 5-ht1b receptor availability in primarily cocaine-dependent humans. *Biological psychiatry*, 76(10):816–822.

[19] Murrough, J. W., Czermak, C., Henry, S., Nabulsi, N., Gallezot, J.-D., Gueorguieva, R., Planeta-Wilson, B., Krystal, J. H., Neumaier, J. F., Huang, Y., et al. (2011a). The effect of early trauma exposure on serotonin type 1b receptor expression revealed by reduced selective radioligand binding. *Archives of general psychiatry*, 68(9):892–900.

[20] Murrough, J. W., Henry, S., Hu, J., Gallezot, J.-D., Planeta-Wilson, B., Neumaier, J. F., and Neumeister, A. (2011b). Reduced ventral striatal/ventral pallidal serotonin 1b receptor binding potential in major depressive disorder. *Psychopharmacology*, 213(2):547–553.

[21] Naganawa, M., Nabulsi, N., Henry, S., Matuskey, D., Lin, S.-F., Slieker, L., Schwarz, A. J., Kant, N., Jesudason, C., Ruley, K., et al. (2021). First-in-human assessment of 11c-lsn3172176, an m1 muscarinic acetylcholine receptor pet radiotracer. *Journal of Nuclear Medicine*, 62(4):553–560.

[22] Neumeister, A., Normandin, M. D., Murrough, J. W.,

Henry, S., Bailey, C. R., Luckenbaugh, D. A., Tuit, K., Zheng, M.-Q., Galatzer-Levy, I. R., Sinha, R., et al. (2012). Positron emission tomography shows elevated cannabinoid cb 1 receptor binding in men with alcohol dependence. *Alcoholism: Clinical and Experimental Research*, 36(12):2104–2109.

[23] Nørgaard, M., Beliveau, V., Ganz, M., Svarer, C., Pinborg, L. H., Keller, S. H., Jensen, P. S., Greve, D. N., and Knudsen, G. M. (2021). A high-resolution in vivo atlas of the human brain's benzodiazepine binding site of gabaa receptors. *NeuroImage*, 232:117878.

[24] Normandin, M. D., Zheng, M.-Q., Lin, K.-S., Mason, N. S., Lin, S.-F., Ropchan, J., Labaree, D., Henry, S., Williams, W. A., Carson, R. E., et al. (2015). Imaging the cannabinoid cb1 receptor in humans with [11c] omar: assessment of kinetic analysis methods, test–retest reproducibility, and gender differences. *Journal of Cerebral Blood Flow & Metabolism*, 35(8):1313–1322.

[25] Pittenger, C., Adams Jr, T. G., Gallezot, J.-D., Crowley, M. J., Nabulsi, N., Ropchan, J., Gao, H., Kichuk, S. A., Simpson, R., Billingslea, E., et al. (2016). Ocd is associated with an altered association between sensorimotor gating and cortical and subcortical 5-ht1b receptor binding. *Journal of affective disorders*, 196:87–96.

[26] Radhakrishnan, R., Matuskey, D., Nabulsi, N., Gaiser, E., Gallezot, J.-D., Henry, S., Planeta, B., Lin, S.-f., Ropchan, J., Huang, Y., et al. (2020). In vivo 5-ht6 and 5-ht2a receptor availability in antipsychotic treated schizophrenia patients vs. unmedicated healthy humans measured with [11c] gsk215083 pet. *Psychiatry Research: Neuroimaging*, 295:111007.

[27] Radhakrishnan, R., Nabulsi, N., Gaiser, E., Gallezot, J.-D., Henry, S., Planeta, B., Lin, S.-f., Ropchan, J., Williams, W., Morris, E., et al. (2018). Age-related change in 5-ht6 receptor availability in healthy male volunteers measured with 11c-gsk215083 pet. *Journal of Nuclear Medicine*, 59(9):1445–1450.

[28] Ranganathan, M., Cortes-Briones, J., Radhakrishnan, R., Thurnauer, H., Planeta, B., Skosnik, P., Gao, H., Labaree, D., Neumeister, A., Pittman, B., et al. (2016). Reduced brain cannabinoid receptor availability in schizophrenia. *Biological psychiatry*, 79(12):997–1005.

[29] Sanchez-Rangel, E., Gallezot, J.-D., Yeckel, C. W., Lam, W., Belfort-DeAguiar, R., Chen, M.-K., Carson, R. E., Sherwin, R., and Hwang, J. J. (2020). Norepinephrine transporter availability in brown fat is reduced in obesity: a human pet study with [11 c] mrb. *International Journal of Obesity*, 44(4):964–967.

[30] Sandiego, C. M., Gallezot, J.-D., Lim, K., Ropchan, J., Lin, S.-f., Gao, H., Morris, E. D., and Cosgrove, K. P. (2015). Reference region modeling approaches for amphetamine challenge studies with [11c] flb 457 and pet. *Journal of Cerebral Blood Flow & Metabolism*, 35(4):623–629.

[31] Sandiego, C. M., Matuskey, D., Lavery, M., McGovern, E., Huang, Y., Nabulsi, N., Ropchan, J., Picciotto, M. R., Morris, E. D., McKee, S. A., et al. (2018). The effect of treatment with guanfacine, an alpha2 adrenergic agonist, on dopaminergic tone in tobacco smokers: An [11 c] flb457 pet study. *Neuropsychopharmacology*, 43(5):1052–1058.

[32] Saricicek, A., Chen, J., Planeta, B., Ruf, B., Subramanyam, K., Maloney, K., Matuskey, D., Labaree, D., Deserno, L., Neumeister, A., et al. (2015). Test–retest reliability of the novel 5-ht 1b receptor pet radioligand [11 c] p943. *European journal of nuclear medicine and molecular imaging*, 42(3):468–477.

[33] Savli, M., Bauer, A., Mitterhauser, M., Ding, Y.-S., Hahn, A., Kroll, T., Neumeister, A., Haeusler, D., Ungersboeck, J., Henry, S., et al. (2012). Normative database of the serotonergic system in healthy subjects using multi-tracer pet. *Neuroimage*, 63(1):447–459.

[34] Slifstein, M., Van De Giessen, E., Van Snellenberg, J., Thompson, J. L., Narendran, R., Gil, R., Hackett, E., Girgis, R., Ojeil, N., Moore, H., et al. (2015). Deficits in prefrontal cortical and extrastriatal dopamine release in schizophrenia: a positron emission tomographic functional magnetic resonance imaging study. *JAMA psychiatry*, 72(4):316–324.

[35] Smart, K., Cox, S. M., Scala, S. G., Tippler, M., Jaworska, N., Boivin, M., Séguin, J. R., Benkelfat, C., and Leyton, M. (2019). Sex differences in [11 c] abp688 binding: a positron emission tomography study of mglu5 receptors. *European journal of nuclear medicine and molecular imaging*, 46(5):1179–1183.

[36] Smith, C. T., Crawford, J. L., Dang, L. C., Seaman, K. L., San Juan, M. D., Vijay, A., Katz, D. T., Matuskey, D., Cowan, R. L., Morris, E. D., et al. (2019). Partial-volume correction increases estimated dopamine d2-like receptor binding potential and reduces adult age differences. *Journal of Cerebral Blood Flow & Metabolism*, 39(5):822–833.

[37] Zakiniaeiz, Y., Hillmer, A. T., Matuskey, D., Nabulsi, N., Ropchan, J., Mazure, C. M., Picciotto, M. R., Huang, Y., McKee, S. A., Morris, E. D., et al. (2019). Sex differences in amphetamine-induced dopamine release in the dorsolateral prefrontal cortex of tobacco smokers. *Neuropsychopharmacology*, 44(13):2205–2211.
