# Supplementary material for: Integrating multimodal and multiscale connectivity blueprints of the human cerebral cortex in health and disease
Source: PLoS Biol. 2023 Sep 25;21(9):e3002314. doi: 10.1371/journal.pbio.3002314 (PMC10553842; doi:10.1371/journal.pbio.3002314)
Supplement: Attachment — Submitted filename: Response to Reviewers.pdf [file pbio.3002314.s011.pdf]

Dear Dr. Smith and Reviewers,

Thank you for the constructive feedback on our first submission, and for the opportunity to revise the manuscript. Following your comments and suggestions, we have thoroughly revised the manuscript. In this letter, we respond to each of the reviewers' comments in detail. Reviewer comments are in **bold font** and our responses are in regular font.

Major changes include: (1) substantially improved scholarship with respect to neuroanatomy and classical literature, including major changes in the Introduction, Results, and Discussion that better contextualize the present findings and clarify novelty (highlighted by Reviewers #1 and #2); (2) clarification of methodology and take-home messages (highlighted by Reviewer #3); and (3) expansion of multiple analyses resulting in three new analyses in the Supplement and one new analysis in Figure 6b (highlighted by Reviewers #2 and #3).

Reviewer #1:

The manuscript entitled "Integrating multimodal, multiscale connectivity blueprints of the cerebral cortex" analyze seven datasets of human cerebral cortex structure and function to, in the words of the authors, "assemble a comprehensive multiscale wiring blueprint of the cerebral cortex". The datasets analyzed comprise gene expression data (Allen Human Brain Atlas), receptor density data (obtained from PET-Scan studies), laminar architecture (BigBrain Atlas), metabolism (glucose uptake estimated by FDG-PET), electrophysiology (Resting state MEG from the Human Connectome Project), and temporal fingerprints (hemodynamic resting state measured by fMRI BOLD form the Human Connectome Project and temporal profile similarity). The authors called these datasets "connectivity modes"; thus, gene expression, receptor density, and laminar architecture are "molecular" or "microscale" connectivity modes whereas hemodynamic connectivity, metabolism, and electrophysiology are "dynamic" connectivity modes.

The major finding of this manuscript is that molecular connectivity modes are well aligned with rich club hubs and selective vulnerability to neuropsychiatric disorders.

I am a classic neuroanatomist with expertise in human and non-human primate brain microscopy, synaptic tract-tracing, and ultrastructure and I cannot judge the conditioning of the data and the statistical procedures used here by the authors, but I do acknowledge that the topic addressed in the present manuscript (the relation of molecular, structural, and functional levels in the human cerebral cortex) is quite interesting for a broad audience of both basic and clinical neuroscientists. Also, I do appreciate the experimental approach of combining different sets of data as productive and convenient. But I do have several serious conceptual concerns that pertain to the misuse of brain terminology, the originality of the identified principles, and the neglect of significant portions of scientific literature on the structure and connections of the human and non-human primate cerebral cortex.

1) Proper use of neuroanatomical terminology: In the title the authors mention the cerebral cortex, but in the abstract and in most places through the text they talk about the brain. According to the Merriam-Webster dictionary, Brain is "the portion of the vertebrate central nervous system enclosed in the skull and continuous with the spinal cord", but it is obvious that the authors have not run data analysis of the thalamus or the striatum: they have analyzed the cerebral cortex. Please, note that the brain is not the cerebral cortex. Brain and cerebral cortex are not interchangeable terms.

We agree with the Reviewer and have corrected the terminology to use “cerebral cortex” or “cortex” instead of “brain” when we mean only the cortex.

2) In search of principles and blueprints: In the abstract, the authors ambitiously declare that they have "uncover a compact set of universal organizational principles whereby all types of inter-regional relationships reflect brain geometry and anatomical connectivity". Also, they state that their work sets nothing less than "the stage for next-generation connectomics and the integrative study of inter-regional relationships". These affirmations by the authors are hyperbolic because, as I will explain below, I could hardly find a single principle through the manuscript that had not been identified before using different techniques.

The major principle that runs through the manuscript is the relation between the laminar architecture of the cerebral cortex and its connections. In the past ten years there has been an explosion of human brain imaging articles in which the structural connectivity between cortical areas was related to "cortical microstructure", "Microscale pattern of cortical organization", "Microanatomy", "microcircuit specialization", "microscale properties", etc. The authors of most of these articles claim to be original in the identification of this principle (the relation between cortical structure and connections), but it was discovered in the 80s by Helen Barbas with synaptic tract-tracing in macaques. Basically, the human and non-human primate cerebral cortex shows a gradient of laminar complexity that is related to the laminar pattern (Barbas 1986; Barbas and Rempel-Clower 1997; García-Cabezas et al. 2019) and the strength (Aparicio-Rodríguez and García-Cabezas 2023) of cortico-cortical connections in a relational model called the Structural Model for Connections. This model allows for predicting the position of cortical areas across cortical hierarchies (Hilgetag and Goulas 2020) and has been used in in vivo MRI human cortex studies (Zhang et al. 2020). Also, it is related to the systematic variation of markers of synaptic plasticity and neuron stability (García-Cabezas et al. 2017; Zikopoulos et al. 2018). Even more, the Structural Model for Connections is compatible with causal mechanism of embryonic development and phylogeny (García-Cabezas et al. 2019; Puelles et al. 2019; García-Cabezas et al. 2022), a fundamental requisite to assert the biological meaning of any proposed principle.

I think that the authors should carefully read the already classic literature of the Structural Model to realize that their findings are just a confirmation of principles identified long ago. The authors find that "across all seven connectivity modes, brain regions that are physically connected by white matter show greater feature similarity than those that are not connected, suggesting that biologically similar neuronal populations are in direct communication" and then claim to have found "that connectivity modes demonstrate common organizational principles that respect geometry, neuroanatomy, and anatomical connectivity, regardless of imaging modality or biological mechanism". In fact, the connectivity modes of the present manuscript just confirm the "organizational principles" that we already knew from neuroanatomical research in human and non-human primates and do not increase our insight on the biological mechanisms underlying the relation between cortical structure and cortico-cortical connections.

We thank the Reviewer for recommending that we integrate neuroanatomical and non-human primate findings in the presentation and interpretation of our results. As will become apparent in this response letter, we have thoroughly revised the manuscript in light of this body of work, in response to Reviewers #1 and #2.

The primary strength and novelty in this study is its integration of multiple perspectives of connectivity, which lets us determine how they are related to one another. We do not study local feature measurements (e.g. laminar differentiation, myelination) at a specific brain region but rather how regions are related to one another with respect to similarities in local phenotypes. We use these similarity matrices (“connectivity modes”) as networks and relate them to each other and to the structural connectome. We do believe that next-generation connectomics will be integrative: for the last decade, macroscale connectomics has focused on the structural connectome and the fMRI-defined functional connectome, which ignore the important local microscale properties deeply intertwined with connectivity (as demonstrated by multiple neuroanatomical studies). Here we show that connectomics is missing out on many other perspectives of “connectivity”, and that these alternative perspectives of connectivity are not redundant but rather tell us something new about cortex organization.

We fully agree that the relationship between laminar similarity and connectivity was already shown in anatomical studies many years ago (Barbas 1986, Barbas & Rempel-Clower 1997), and apologize for our neglect of this body of scientific literature. Indeed the relationship between feature similarity and connectivity has been shown for multiple connectivity modes (Fornito et al 2019, Hansen et al 2022, Hagmann et al 2008, Shafiei et al 2020). Figure 1 shows that all connectivity modes, regardless of biological mechanism or imaging modality, present these fundamental organization properties. This sets the stage for the remainder of the study where we focus on the unique ways in which connectivity modes reflect brain organization. We agree that the use of the word “principle” exaggerates the importance and novelty of the commonalities; we have therefore removed this terminology.

First, we remove hyperbolic claims in the Abstract:

> “The brain is composed of disparate neural populations that communicate and interact with one another. Although fiber bundles, similarities in molecular architecture, and synchronized neural activity all reflect how brain regions potentially interact with one another, a comprehensive study of how all these inter-regional relationships jointly reflect brain structure and function remains missing. Here we systematically integrate seven multimodal, multiscale types of inter-regional similarity (“connectivity modes”) derived from gene expression, neurotransmitter receptor density, cellular morphology, glucose metabolism, haemodynamic activity, and electrophysiology. ~~We uncover a compact set of universal organizational principles whereby all types of inter-regional relationships reflect geometry and anatomical connectivity.~~ We first show that for all connectivity modes, feature similarity decreases with distance and increases when regions are structurally connected. Next we show that connectivity modes exhibit unique and diverse connection patterns, hub profiles, spatial gradients, and modular organization. Throughout, we observe a consistent primacy of molecular connectivity modes---namely correlated gene expression and receptor similarity---that map onto multiple phenomena, including the rich club and patterns of abnormal cortical thickness across 13 neurological, psychiatric, and neurodevelopmental disorders. Finally, to construct a single multimodal wiring map of the cortex, we fuse all seven connectivity modes and show that the fused network maps onto major organizational features of the cortex including structural connectivity, intrinsic functional networks, and cytoarchitectonic classes. Altogether, this work contributes to ~~sets the stage for next-generation connectomics and~~ the integrative study of inter-regional relationships.”

Next, we revise the Introduction to include a new paragraph about findings from neuroanatomical work. This serves as a primer as neuroanatomical studies are now integrated throughout the Results and Discussion.

("Introduction" section, Paragraph #2):

> "However, the graph representation of the structural connectome, in which regional nodes are identical, does not account for the molecular and physiological heterogeneity that exists in the brain. An emerging representation of connectivity is feature similarity: if two brain regions exhibit similar biological features, we might expect them to be related to one another and engaged in common function (Zilles et al 2015, Anderson et al 2018, Paquola et al 2019, Voigt et al 2022, Horwitz et al 1984). Neuroanatomical tract-tracing studies in non-human primates have extensively shown that biological feature similarity is fundamental to brain organization (Barbas 1986, Garcia-Cabezas et al 2019). These pioneering studies demonstrated that neuronal projection patterns can be predicted based on the laminar differentiation of the source and target regions (Barbas & Rempel-Clower 1997), and has been extended to human prefrontal cortex and other model organisms (Zikopoulos et al 2018, Goulas et al 2019, Beul et al 2015). Furthermore, local differences in laminar architecture follow a gradient of receptor density (Goulas et al 2021, Hansen et al 2022), and synaptic plasticity (Garcia-Cabezas et al 2017), indicating an alignment between multiple local features and connectivity. However, these studies are currently limited to qualitative measurements of cytoarchitectonic similarity, small subsets of brain regions, model organisms, and to a single perspective of molecular make-up."

We also revise the first subsection about common organizational patterns of connectivity modes to appropriately reference neuroanatomical work.

("Results" section, "Common organizational patterns of connectivity modes" subsection, Paragraph #3):

> "We next sought to relate each connectivity mode to the brain's underlying structural architecture. We constructed a weighted structural connectome using diffusion-weighted MRI data from the Human Connectome Project; this network represents whether, and how much, two cortical regions are connected by white matter streamlines. We find that, across all seven connectivity modes, cortical regions that are physically connected by white matter show greater feature similarity than those that are not connected (Fig. 1c). These differences are greater than in a population of degree- and edge length-preserving surrogate structural connectomes, indicating that the effect is specifically due to wiring rather than spatial proximity (Betzel et al 2018). Notably, neuroanatomical studies in model organisms have found that cytoarchitectonic similarity predicts neuronal projections better than distance (Beul et al 2015, Beul et al 2017, Aparicio-Rodriguez et al 2023); we expand on this by showing that all connectivity modes, from gene expression to neural dynamics, demonstrate greater similarity for structurally connected cortical regions in the human. Finally, for the subset of edges with a structural connection, we find a correlation between the strength of the structural connection and each connectivity mode's edge weight (Fig. 1d) (Honey et al 2009, Aparicio-Rodriguez et al 2023). Altogether, we find that connectivity modes demonstrate commonalities that respect distance, neuroanatomy, and anatomical connectivity, regardless of imaging modality or biological mechanism."

Altogether, these changes clarify the ways in which our work extends the Structural Model and tells us something new about brain organization, namely:

1. While the Structural Model presents a theory of synaptic connectivity according to laminar differentiation, we look at multiple phenotypes from genes, receptors, lamination, metabolism, and dynamics (fMRI, MEG, and temporal feature fingerprints). Importantly, our work is not a theory of cortical structural connectivity. We use structural connectivity in the first figure to show that all connectivity modes, despite their diverse biological mechanisms, follow similar organizational patterns according to distance and structural connectivity. The rest of the study is about how we can generate new networks based on multiscale phenotypes to make new discoveries about brain organization.

2. These new discoveries include:
    a. There is a dichotomy between how molecular and dynamic features map onto structural connectivity, which to our knowledge has not been tested before (Figure 2b, Figure 2d). This highlights the importance of studying multi-scale perspectives of connectivity (i.e. integrative connectomics).
    b. Each connectivity mode shows a unique hub profile, gradient decomposition, and modular organization. Much of the work in the neuroimaging field on hubs, gradients, and modules has focused on single “template” descriptions of hubs/gradients/modules, or on how correlated hubs/gradients/modules are across different brain phenotypes. We show that each level of description has a distinct organization which should be explicitly considered in future work.
    c. By treating feature similarity as networks, we can predict disease-specific cortical structural abnormalities. Importantly, genetic and receptor fingerprints perform better than wiring and distance.
    d. Using a network fusion algorithm, we can combine all networks into a single network that maximizes the alignment with structural connectivity, intrinsic functional networks, and cytoarchitectonic classes.

3. While the Structural Model tells us about connectivity at the microscale (projections between neurons), and primarily in non-human primates, our work extends to the macroscale (large white-matter bundles between spatially segregated brain regions) in in-vivo humans.

Barbas H (1986) Pattern in the laminar origin of corticocortical connections. J Comp Neurol 252 (3):415-422. doi:10.1002/cne.902520310

Barbas H, Rempel-Clower N (1997) Cortical structure predicts the pattern of corticocortical connections. Cereb Cortex 7 (7):635-646

García-Cabezas MA, Zikopoulos B, Barbas H (2019) The Structural Model: A theory linking connections, plasticity, pathology, development and evolution of the cerebral cortex. Brain structure & function 224 (3):985-1008. doi:10.1007/s00429-019-01841-9

Fornito, A., Arnatkevičiūtė, A., & Fulcher, B. D. (2019). Bridging the gap between connectome and transcriptome. *Trends in cognitive sciences*, *23*(1), 34-50.

Hansen, J. Y., Shafiei, G., Markello, R. D., Smart, K., Cox, S. M., Nørgaard, M., ... & Misic, B. (2022). Mapping neurotransmitter systems to the structural and functional organization of the human neocortex. *Nature Neuroscience*, 1-13.

Hagmann, P., Cammoun, L., Gigandet, X., Meuli, R., Honey, C. J., Wedeen, V. J., & Sporns, O. (2008). Mapping the structural core of human cerebral cortex. *PLoS biology*, *6*(7), e159.

Shafiei, G., Markello, R. D., Vos de Wael, R., Bernhardt, B. C., Fulcher, B. D., & Misic, B. (2020). Topographic gradients of intrinsic dynamics across neocortex. *eLife*, *9*, e62116.

There are other places in the text where the authors claim to have identified a principle and try to relate it to biology. For instance, the authors find that the "edges in the brain's topological rich club regime are particularly dominated by molecular features (e.g. laminar similarity, correlated gene expression, and receptor similarity" and conclude that these results point to the possible biological origins of the rich club. Namely, the rich club may reflect coordinated patterns of inter-regional microscale similarity". The affirmation that these results point at the possible biological origins of the rich club or of any other structural or functional feature of the cerebral cortex needs a (at least partial) causal explanation, otherwise what the authors have found is not a principle but mere correlation without clear biological meaning. And in biology, causal mechanisms must be searched for in ontogeny and phylogeny.

We have removed language about implied causality as this analysis does not make clear whether the rich club results in molecular similarity, or if molecular similarity results in the rich club (or both!).

("Results" section, "Structural and geometric features of connectivity modes" subsection, Paragraph #5):

> "We find that edges in the cortex's topological rich club regime are particularly dominated by molecular features (e.g. laminar similarity, correlated gene expression, and receptor similarity) (Beul et al 2015). Haemodynamic and electrophysiological connectivity are especially weak for links between high-degree regions, and temporal similarity is unstable. Metabolic connectivity is an additional connectivity mode that demonstrates significantly increased edge strength for links between high-degree regions, suggesting that energy consumption is synchronized between structural hubs (Fulcher et al 2016, Arnatkeviciute et al 2021, Liang et al 2013, Vaishnavi et al 2010). Collectively, these ~~results point to the biological origins of the rich club~~ findings indicate that the rich club may reflect coordinated patterns of inter-regional microscale similarity across multiple molecular features. On the other hand, the rich club is not characterized by similar neural dynamics, possibly related to the functional flexibility of these regions (Griffa et al 2018)."

Finally, at the end of the Discussion, the authors affirm that "The consistent primacy of molecular connectivity modes demonstrates that mapping brain connectivity from the perspective of the underlying biology—gene transcription, receptor density, cellular composition—is just as, if not more, informative than oft-studied dynamical modes such as haemodynamic connectivity". So underlying biology matters in human cortex neuroscience. Is this a surprising conclusion?

Given the fundamental importance of molecular organization in human cortex neuroscience, it is indeed surprising that the field of cortical connectomics has been slow to adopt multiscale perspectives of connectivity that include the underlying biology. We are excited by our finding that macroscale whole-cortex networks on inter-regional relationships—traditionally only studied from the perspective of

white-matter bundles or correlated fMRI BOLD activity—can be represented from the perspective of molecular feature similarity to tell us something new about brain organization. In fact these molecular connectivity modes map onto known architectural features better than oft-studied dynamic modes and can explain disease vulnerability better than structure, distance, and dynamics. This study presents the value in studying whole-cortex connectivity from multiple perspectives and will hopefully inspire future connectomics studies to do the same.

We have clarified the sentence in the Discussion to explicitly state “microscale features” rather than “biology”, which was too vague (“Discussion” section, Paragraph #8):

> “The consistent primacy of molecular connectivity modes demonstrates that mapping cortical connectivity from the perspective of ~~the underlying biology~~ underlying microscale features—gene transcription, receptor density, cellular composition—is just as, if not more, informative than oft-studied dynamical modes such as haemodynamic connectivity.”

3) Neglect of classic scientific literature: At several places the authors seem to ignore relevant and fundamental pieces of scientific literature. It is like if they only knew about contemporary brain imaging articles; but contemporary neuroscience resulted from the integration of multiple approaches in multiple species. Neuroscientists of the XXIst century must be specialist in a given field of brain research but they should also be aware of fundamental knowledge from other fields.

For example, a significant finding in the present manuscript is the relation between molecular connectivity modes and selective vulnerability to neuropsychiatric disorders. The authors say that "Emerging theories emphasize that the course and expression of multiple brain diseases is mediated by shared molecular vulnerability [61, 169]". But this is far from being an emerging theory. Actually, Oskar and Cécile Vogt were the first to advance the concept of selective vulnerability and related it to differences in the physicochemical composition of neurons across brain regions. The authors can read the informed review of Klatzo (2003) to learn about the origin of selective vulnerability as a concept.

Indeed the main text of the manuscript was written primarily from the perspective of human brain imaging and connectomics research. We agree with the Reviewer that providing the historical context for these analyses, as well as a broader view of the neuroscientific literature would strengthen the manuscript.

We have modified the paragraph in question to highlight the historical context of this work and clarify that the emerging theories to which we refer are highlighting the role of multiple rather than a single molecular phenotype on disease progression over the structural connectome.

(“Results” section, “Connectivity modes and disease-specific abnormal cortical thickness” subsection, Paragraph #1):

> “Pioneering studies in post-mortem tissue gave rise to the theory that the physicochemical composition of neurons at local brain regions results in a selective vulnerability to brain disease (Klatzo 2003). Other classical studies have shown that disease propagation in the cerebral cortex is related to microscale features such as myelination (Braak & Braak 1996). These propagation patterns have been successfully modeled at the level of the whole-cortex using the structural connectome, and often perform better when informed by local biological features such as the expression of a

specific gene (Henderson et al 2019, Shafiei et al 2023). Recent findings build on this notion and posit that the course and expression of multiple brain diseases on the structural connectome is mediated by multiple forms of molecular vulnerability rather than a single molecular perturbation (Warren et al 2013, Hansen et al 2022). We therefore tested whether disease propagation patterns derived from the connectivity modes could predict abnormal cortical thickness patterns for thirteen different neurological, psychiatric, and neurodevelopmental diseases and disorders from the ENIGMA consortium (N=21,000 patients, N=26,000 controls) (Thompson 2020, Lariviere et al 2021, Hansen et al 2022). The disease-specific abnormal cortical thickness patterns are regional z-scored case-versus-control effect sizes, representing deviation from normative cortical thickness. We refer to these regional values as “abnormal cortical thickness” or simply “abnormality”.”

Also, the authors affirm that "This suggests that brain regions with similar molecular makeup may undergo similar structural changes in disease". Again, the idea of different vulnerabilities across cortical regions in the human brain was already advanced and exemplified for Alzheimer´s disease by Braak and Braak (1996).

We thank the Reviewer for highlighting this sentence as we have come to realize it is a red herring in the paragraph and should be removed. The novelty in this analysis and the ensuing findings are that structural changes in disease can be predicted better by a network defined by similarities in gene expression and receptor density rather than white-matter connectivity or distance. Furthermore, we make these conclusions based on findings from 13 different diseases and disorders which suggests that similarities in gene expression and receptor density are fundamental to the course of pathology. Although pioneering work has demonstrated that regional properties (e.g. myelination) are related to regional vulnerabilities to specific diseases, we extend this to find the specific properties whose *similarity* (not simply local features but how these features reflect features in other regions) amplifies disease expression in a disease-general manner. Note that we now cite Braak and Braak 1996 in the first paragraph of this subsection (see our response to the comment above).

The paragraph now reads (“Results” section, “Connectivity modes and disease-specific abnormal cortical thickness” subsection, Paragraph #3):

> “We find that correlated gene expression and receptor similarity most consistently amplify the exposure of pathology in a manner that closely resembles the structural cortical profile of the disease. Interestingly, when we repeat the analysis using only negative edges (c_{ij} if $c_{ij} < 0$), we find opposite results: brain regions with high abnormality are negatively connected (that is, are dissimilar to) regions with low abnormality (Figure S5). This suggests that dissimilarity may attenuate disease spread. By repeating the analysis using weighted structural connectivity (in which case we only consider structurally-connected regions) and Euclidean distance between brain regions (in which case we always consider the full network), we are able to uncover cases where feature similarity amplifies disease exposure more than structure or distance alone (Figure 4c). Abnormal cortical thickness patterns of psychiatric disorders in particular (e.g. MDD, schizophrenia, bipolar disorder, OCD) are better explained by correlated gene expression and receptor similarity than structure or distance. This integrative analysis makes it possible to zero in on the imaging modalities and biological mechanisms that reflect cortical pathology in a disease-general manner. Furthermore, it demonstrates the value in

employing feature similarity as a network rather than limiting network models to the structural connectome."

Another example of classic and fundamental literature neglect is found in the Discussion where the authors state that "A second assumption, this stemming primarily from fMRI studies, is that the brain ubiquitously follows a unimodal-transmodal hierarchical gradient, and can be organized in terms of intrinsic resting-state networks". This is not emerging primarily from fMRI studies. On the contrary, it goes back to fundamental physiological and anatomical primate research done in the 60s, 70s, and 80s. I just recommend to the authors carefully reading the well-known article of Mesulam (1998).

We fully agree with the Reviewer that the presence of a unimodal-transmodal hierarchical gradient was not introduced by fMRI studies and apologize for the poorly worded sentence - indeed, the theory was already well established many years ago (as is evident by the Mesulam 1998 review article and also by early work on the subject including Jones and Powell 1970 and Pandya and Kuypers 1969). Our intent for this paragraph is to highlight the fact that the prominence of fMRI "functional connectivity" in the field of connectomics has resulted in a series of assumptions about brain architecture. We aimed to show the field that not all cortical features are aligned with these functionally-defined hierarchies or communities. Specifically, we show that principal gradients (i.e. the first principal component of a matrix) do not all follow a unimodal-transmodal hierarchy (as it does in fMRI).

We have reworded this section and cite the influential Mesulam 1998 paper ("Discussion" section, Paragraph #5):

> "The study of connectomics has been dominated by a focus on structural and haemodynamic connectivity. This has resulted in the assumption that, at the level of the whole-cortex, homologous (Roberts et al 2016), spatially proximal (Fornito et al 2019), and structurally connected (Hilgetag et al 2019) brain regions tend to be more similar. By systematically integrating seven multi-scale perspectives of cortical connectivity, we can conclude in a more systematic and comprehensive manner that these properties are indeed fundamental to cortical organization but that there is considerable variation across connectivity modes. For example, the negative exponential relationship with distance is almost linear for molecular connectivity modes, especially when we consider geodesic instead of Euclidean distance (Figure S2). A second assumption is that diverse brain features should all follow the functionally-defined unimodal-transmodal hierarchical gradient, and can be organized in terms of intrinsic resting-state networks (Mesulam 1998, Jones & Powell 1970, Pandya & Kuypers 1969, Margulies et al 2016, Yeo & Krienen et al 2011). However, we find that microscale connectivity modes (e.g. correlated gene expression, receptor similarity) are well delineated by a partition based on cytoarchitectonic classes whereas dynamic connectivity modes (e.g. haemodynamic connectivity, electrophysiological connectivity) fit better into intrinsic functional systems. Indeed, connectivity modes are poorly correlated with one another, suggesting that each connectivity mode provides a fundamentally different but important view of how cortical regions participate in neural circuits at different spatial and temporal scales (Bazinet et al 2021)."

4) Neuroscience is not only about human brain imaging: I invite the authors to reconsider their statements about connectomics as the "increasingly becoming the dominant paradigm in neuroscience [13, 88, 145]". I invite them to explore the classical human and non-human

neuroanatomy and neurophysiology literature, but also other contemporary paradigms, like those emanating from the developmental biology of the brain [e. g., Nieuwenhuys and Puelles (2016); Nieuwenhuys (2017)], that are changing our views of the human brain in many ways that are much deeper than in vivo brain imaging.

We agree with the Reviewer that studying brain connectivity outside of human imaging has been a dominant paradigm for many years, and that there are other ways to study brain organization and connectivity that human imaging. We apologize for the dismissive language as connectomics is certainly not becoming *the* dominant paradigm in neuroscience, rather just one important paradigm in neuroscience. We have updated this sentence in the text (“Discussion” section, Paragraph #2):

> “Connectomics---the study of relationships between neural elements across multiple scales---is an important and popular paradigm in neuroscience (Bassett & Sporns 2017, Sporns et al 2005, Lichtman et al 2011).”

Furthermore, we have added a new paragraph to the Discussion where we zoom out of the human neuroimaging field to discuss findings about brain connectivity from classical as well as more recent neuroanatomical studies.

(“Discussion” section, Paragraph #10):

> “Throughout this report, we have illustrated how cortical connectivity can be extended from neural wiring to many complementary perspectives of inter-regional relationships. We focus on densely sampled data across the whole-cortex to make general claims about patterns of cortical organization. Most of the data employed are derived from *in vivo* neuroimaging in relatively large samples of healthy adult humans. However, these questions about multiscale cortical organization can---and have, for decades---be asked with more anatomical specificity using methods such as cell staining and tract-tracing in small samples of *ex-vivo* brains, generally from model organisms (Zilles et al 1988, Barbas 1986, Garcia-Cabezas et al 2019). Such studies have demonstrated that neurons make projection patterns that are tightly linked to the cellular architecture of the cortex, including the laminar differentiation of the source and target of a neural projection (Barbas & Rempel-Clower 1997) (e.g. the Structural Model, reviewed in Garcia-Cabezas et al 2019). Intertwined with laminar differentiation and tract-tracing projection patterns is also the phylogenetic age of the cortical region (Garcia-Cabezas et al 2022), markers of plasticity and stability (Garcia-Cabezas et al 2017), and likely also receptor architecture (Palomero et al 2019, Froudist-Walsh et al 2023). Furthermore, the field of developmental biology presents fundamental organizational principles for how the brain develops its spatial organization and topology (Nieuwenhuys 2017). Large-scale neuroimaging connectomic studies complement biological and neuroanatomical studies by extending predictions to the scale of the whole human cortex and across many more brain phenotypes. For example we confirm that laminar similarity is related to connectivity and the brain's rich club (Beul et al 2015), but extend this to gene expression, receptor architecture, and metabolism. The synergy between neuroanatomical and imaging fields is necessary to fully capture inter-regional relationships across multiple layers of description.”

Miguel Ángel García-Cabezas.

References

Aparicio-Rodríguez G, García-Cabezas MÁ (2023) Comparison of the predictive power of two models of cortico-cortical connections in primates: The Distance Rule Model and the Structural Model. Cereb Cortex Online ahead of print. doi:doi: 10.1093/cercor/bhad104

Barbas H (1986) Pattern in the laminar origin of corticocortical connections. J Comp Neurol 252 (3):415-422. doi:10.1002/cne.902520310

Barbas H, Rempel-Clower N (1997) Cortical structure predicts the pattern of corticocortical connections. Cereb Cortex 7 (7):635-646

Braak H, Braak E (1996) Development of Alzheimer-related neurofibrillary changes in the neocortex inversely recapitulates cortical myelogenesis. Acta Neuropathol 92 (2):197-201. doi:10.1007/s004010050508

García-Cabezas MA, Hacker JL, Zikopoulos B (2022) Homology of neocortical areas in rats and primates based on cortical type analysis: an update of the Hypothesis on the Dual Origin of the Neocortex. Brain structure & function Online ahead of print. doi:doi.org/10.1007/s00429-022-02548-0

García-Cabezas MA, Joyce MKP, John YJ, Zikopoulos B, Barbas H (2017) Mirror trends of plasticity and stability indicators in primate prefrontal cortex. Eur J Neurosci 46 (8):2392-2405. doi:10.1111/ejn.13706

García-Cabezas MA, Zikopoulos B, Barbas H (2019) The Structural Model: A theory linking connections, plasticity, pathology, development and evolution of the cerebral cortex. Brain structure & function 224 (3):985-1008. doi:10.1007/s00429-019-01841-9

Hilgetag CC, Goulas A (2020) 'Hierarchy' in the organization of brain networks. Philos Trans R Soc Lond B Biol Sci 375 (1796):20190319. doi:10.1098/rstb.2019.0319

Klatzo I (2003) Cecile & Oskar Vogt: the significance of their contributions in modern neuroscience. Acta Neurochir Suppl 86:29-32. doi:10.1007/978-3-7091-0651-8_6

Mesulam MM (1998) From sensation to cognition. Brain 121:1013-1052

Nieuwenhuys R (2017) Principles of Current Vertebrate Neuromorphology. Brain Behav Evol 90 (2):117-130. doi:10.1159/000460237

Nieuwenhuys R, Puelles L (2016) Towards a New Neuromorphology. doi:10.1007/978-3-319-25693-1

Puelles L, Alonso A, Garcia-Calero E, Martinez-de-la-Torre M (2019) Concentric ring topology of mammalian cortical sectors and relevance for patterning studies. J Comp Neurol 527 (10):1731-1752. doi:10.1002/cne.24650

Zhang J, Scholtens LH, Wei Y, van den Heuvel MP, Chanes L, Barrett LF (2020) Topography impacts topology: Anatomically central areas exhibit a "High-Level Connector" profile in the human cortex. Cereb Cortex 30 (3):1357-1365. doi:10.1093/cercor/bhz171

Zikopoulos B, García-Cabezas MA, Barbas H (2018) Parallel trends in cortical gray and white matter architecture and connections in primates allow fine study of pathways in humans and reveal network disruptions in autism. PLoS Biol 16 (2):e2004559. doi:10.1371/journal.pbio.2004559

Reviewer #2:

The paper entitled "Integrating multimodal, multiscale connectivity blueprints of the cerebral cortex" is a first effort to systematically compare and bring together distinct types of inter-regional similarity estimates that can and have been used to describe the organization of the cerebral cortex and its connectome. The findings suggest that all types of similarity estimates reflect more or less inter-regional topology and connectivity, which emerge as universal organizational principles of the cortex. Importantly, the authors also found unique relationships that emerge from the comparison of distinct types of inter-regional similarity estimates and highlight distinct associations between them. To this point, the authors highlight the variability of hubness profiles across modalities, which points out the importance of characterizing network architecture from multiple complementary perspectives.

This is a very timely, interesting, and well written study and I want to commend the authors on their effort, care, and consideration of relevant literature, while completing this much-needed work. The strengths of this manuscript include (1) the impressive integration of seven multimodal and multiscale types of inter-regional cortical similarity, (2) the rigorous approach used to analyze and compare these seven modes, including sensitivity and replication analysis using multiple parcellation schemes, and (3) the development of a framework that can be used to correlate data from disparate sets of variables at multiple scales.

The main limitations arise from compiling data for each modality across age, sex, and other key factors, like handedness (not mentioned in this article), limitations of each dataset used e.g., non-specific binding in PET datasets, and challenges when comparing inherently different types of data. Most were addressed adequately by the authors. As the authors stated, all limitations can be addressed in future in-depth studies, as new datasets become available for each feature used to determine these inter-regional similarity estimates however, I would clarify that they can also be addressed by concurrently using multiple datasets for each type of similarity estimate that are currently available. The authors addressed transmodal comparison challenges through an elegant study design that included alternative parcellation schemes, rank-transformations, and normalization of data. Therefore, despite the limitations, the findings are novel, highly significant, and the manuscript is poised to be an outstanding contribution to our field and of general interest.

Below few comments and suggestions that in my opinion will further increase the value and clarity of the manuscript, improve presentation, and strengthen reported findings and discussion:

1) In the introduction (first paragraph), the authors mention the prevalence of short-range connections that result in functionally segregated modules, as a hallmark feature of the structural architecture of the brain. A recent elegant study by Rosen and Halgren, PLOS Biology 2022 provided key evidence for the sparse connectivity in the human brain and should be cited here.

We agree with the Reviewer and have cited Rosen & Halgren 2022 in the Introduction:

> "Across organisms, spatial scales, and imaging techniques, the brain's white-matter architecture, also called "structure", exhibits hallmark features including a prevalence of short range connections resulting in functionally segregated modules (Sporns et al 2016, Rosen & Halgren 2022), and a small number of disproportionately densely interconnected hubs (van den Heuvel & Sporns 2011)."

2) The sentence starting the second paragraph is not entirely accurate and can be misleading leading to gaps in the presentation of the relationship between structure and function of the cortex presented in the second and third paragraphs. Several studies have shown that structure can account for key molecular and functional attributes of the brain, especially in the cortex. For example, Goulas et al., PNAS 2021 showed a receptor-based natural axis of the human cerebral cortex, which also parallels laminar architecture; Garcia-Cabezas et al., European Journal Of Neuroscience 2017 showed that cortical structure is systematically associated with several plasticity and stability markers; Garcia-Cabezas et al., Brain Structure and Function 2022 and Goulas et al., PLOS Biology 2019 showed how the organization of cortical structure and connectivity are rooted in the development and can be seen throughout evolution.

We agree with the Reviewer and apologize for the unclear sentence. Our intent was to highlight the fact that the structural connectome—the set of nodes (brain regions) and edges (white matter tracts, measured using diffusion MRI in vivo)—generally assumes that all nodes are homogeneous and therefore does not account for the rich biological heterogeneities that exist in the brain. Indeed, as the Reviewer points out, these heterogeneities (receptor densities, cytoarchitecture, etc) are deeply intertwined with both regional structure (e.g. laminar differentiation) as well as structural connectivity and should therefore be considered when we study the structural connectome as a network.

Note that in the present report, we use "structure" to refer to the white-matter architecture of the brain, rather than the structural composition (e.g. laminar differentiation) of specific regions. We therefore cite Goulas et al 2021, Garcia-Cabezas et al 2017, Garcia-Cabezas et al 2022, and Goulas et al 2019 later in the Introduction (shown also in our response to the next comment and to Reviewer #1).

("Introduction" section, Paragraph #1):

> "Across organisms, spatial scales, and imaging techniques, the brain's white-matter architecture ("structure") exhibits hallmark features including a prevalence of short range connections resulting in functionally segregated modules (Sporns et al 2016, Rosen & Halgren 2022), and a small number of disproportionately densely interconnected hubs (van den Heuvel & Sporns 2011)."

("Introduction" section, Paragraph #2):

> "However, the graph representation of the structural connectome, in which regional nodes are identical, does not account for the molecular and physiological heterogeneity that exists in the brain. An emerging representation of connectivity is feature similarity: if two brain regions exhibit similar biological features, we might expect them to be related to one another and engaged in common function (Zilles et al 2015, Anderson et al 2018, Paquola et al 2019, Voigt et al 2022, Horwitz et al 1984). Neuroanatomical tract-tracing studies in non-human primates have extensively shown that biological feature similarity is fundamental to brain organization (Barbas 1986, Garcia-Cabezas et al 2019). These pioneering studies demonstrated that neuronal projection patterns can be predicted based on the laminar differentiation of the source and target regions (Barbas & Rempel-Clower 1997), and has been extended to human prefrontal cortex and other model organisms (Zikopoulos et al 2018, Goulas et al 2019, Beul et al 2015). Furthermore, local differences in laminar architecture follow a gradient of receptor density (Goulas et al 2021, Hansen et al 2022), and synaptic plasticity (Garcia-Cabezas et al 2017), indicating an alignment between multiple local features and connectivity. However,

> these studies are currently limited to qualitative measurements of cytoarchitectonic similarity, small subsets of brain regions, model organisms, and to a single perspective of molecular make-up."

3) The first part of the second paragraph of the introduction introduces key principles of the organization of the cortex including the fact that the most common connections are between similar areas, something that is true for primates and likely all mammals, extensively presented and discussed in several studies e.g., Garcia-Cabezas et al., Brain Structure and Function 2019.

We fully agree with the Reviewer and have thoroughly revised the manuscript to contextualize our findings within the broad neuroanatomical and non-human primate literature. This includes a new paragraph in the Introduction ("Introduction" section, Paragraph #2; already shown in our response to the previous comment):

> "However, the graph representation of the structural connectome, in which regional nodes are identical, does not account for the molecular and physiological heterogeneity that exists in the brain. An emerging representation of connectivity is feature similarity: if two brain regions exhibit similar biological features, we might expect them to be related to one another and engaged in common function (Zilles et al 2015, Anderson et al 2018, Paquola et al 2019, Voigt et al 2022, Horwitz et al 1984). Neuroanatomical tract-tracing studies in non-human primates have extensively shown that biological feature similarity is fundamental to brain organization (Barbas 1986, Garcia-Cabezas et al 2019). These pioneering studies demonstrated that neuronal projection patterns can be predicted based on the laminar differentiation of the source and target regions (Barbas & Rempel-Clower 1997), and has been extended to human prefrontal cortex and other model organisms (Zikopoulos et al 2018, Goulas et al 2019, Beul et al 2015). Furthermore, local differences in laminar architecture follow a gradient of receptor density (Goulas et al 2021, Hansen et al 2022), and synaptic plasticity (Garcia-Cabezas et al 2017), indicating an alignment between multiple local features and connectivity. However, these studies are currently limited to qualitative measurements of cytoarchitectonic similarity, small subsets of brain regions, model organisms, and to a single perspective of molecular make-up."

The first part of the second paragraph in the Introduction is specifically about similar biological make-up being related to common function, not necessarily physical connectivity. We therefore add citations to studies relating biological feature similarity to common function here in the Introduction (also in response to Reviewer #3 Comment #11). We include references to neuroanatomical studies (e.g. reviewed by Garcia-Cabezas et al 2019) when we present key principles of cortex organization and connectivity in the Results as well as in the Discussion (see our response to Reviewer #2 Comment #6).

("Introduction" section, Paragraph #2):

> "An emerging representation of connectivity is feature similarity: if two brain regions exhibit similar biological features, we might expect them to be related to one another and engaged in common function (Zilles et al 2015, Anderson et al 2018, Paquola et al 2019, Voigt et al 2022, Horwitz et al 1984)."

4) The use of the gwMRF parcellation of the cortex into 400 regions for the main analysis is first mentioned in the results and then in several other segments of the paper however, it is not clear

why this parcellation scheme was selected for the main, detailed analysis over other commonly used schemes. More information on this and potential confounding effects based of the parcellation schemes used would be useful. Since there is little agreement between several, if not most, parcellation schemes, one could ask if the approach used in this paper could form the basis for a more general/universal parcellation of the cortex.

We selected the functionally-defined Schaefer parcellation for multiple reasons: (1) it has been shown to respect both functional and architectonic cortical boundaries, (2) the parcels are approximately equally sized, (3) we were concerned about using an anatomical parcellation defined based on sulcal and gyral landmarks because many of the presently studied features exhibit large variability within single gyri, and (4) it optimizes within-parcel functional homogeneity better than other previously proposed functionally-defined parcellations (e.g. Craddock et al 2012, Shen et al 2013, Glasser et al 2016, Gordon et al 2016). However, since this parcellation is defined using functional MRI, it's possible that the functional connectivity modes (especially haemodynamic connectivity) are better represented by this parcellation than the molecular connectivity modes. To mitigate this, we repeated the analyses using the Desikan-Killiany parcellation, which is defined anatomically according to sulci and gyri (Supplementary Figure S7).

We have added this motivation to the Methods section where we introduce the parcellation ("Methods" section, "Connectivity modes" subsection, Paragraph #1):

> "Each connectivity mode is defined across 400 cortical cortical regions, ordered according to 7 intrinsic networks (visual, somatomotor, dorsal attention, ventral attention, limbic, frontoparietal, default mode), separated by hemispheres (left, right) (Schaefer et al 2018). This functionally-defined parcellation scheme was chosen because the parcels are approximately equal in size and parcel boundaries respect both functional boundaries (as determined by resting-state and task-based fMRI) as well as histological boundaries (Schaefer et al 2018). Nonetheless, we repeated the analyses using the coarser 100-region Schaefer parcellation as well as an anatomically-defined 68-region Desikan Killiany parcellation and found consistent results (Fig S7; parcellated connectivity modes all available at https://github.com/netneurolab/hansen_many_networks)."

We have also added a point to the limitations about the Schaefer parcellation potentially biasing results towards functional connectivity modes. The Reviewer's idea about the development of a multimodal, multi-scale parcellation is an exciting (and very plausible!) idea for future research.

("Discussion" section, Paragraph #11):

> "Fifth, the chosen functionally-defined Schaefer parcellation used for all main analyses may better reflect functional networks (e.g. haemodynamic connectivity, electrophysiological connectivity, temporal similarity) than molecular networks. We aimed to mitigate this limitation by repeating analyses using an anatomically-defined parcellation (Desikan-Killiany; Supplementary Figure S7). Future integrative parcellations designed using multiple brain phenotypes would be ideal for studying multi-scale, multi-modal connectivity modes."

Craddock, R. C., James, G. A., Holtzheimer III, P. E., Hu, X. P., & Mayberg, H. S. (2012). A whole brain fMRI atlas generated via spatially constrained spectral clustering. *Human brain mapping*, *33*(8), 1914-1928.

Shen, X., Tokoglu, F., Papademetris, X., & Constable, R. T. (2013). Groupwise whole-brain parcellation from resting-state fMRI data for network node identification. *Neuroimage*, *82*, 403-415.

Glasser, M. F., Coalson, T. S., Robinson, E. C., Hacker, C. D., Harwell, J., Yacoub, E., ... & Van Essen, D. C. (2016). A multi-modal parcellation of human cerebral cortex. *Nature*, *536*(7615), 171-178.

Gordon, E. M., Laumann, T. O., Adeyemo, B., Huckins, J. F., Kelley, W. M., & Petersen, S. E. (2016). Generation and evaluation of a cortical area parcellation from resting-state correlations. *Cerebral cortex*, *26*(1), 288-303.

5) In Results, page 2 the authors mention "Homotopic connections stand out, indicating that homologous brain regions in left and right hemispheres are consistently similar to each other no matter the biological feature". Relevant to this and other statements about connections involving one or both hemispheres, previous work in non-human primates has linked structure and connections in the cortex and showed parallel organization of contralateral and ipsilateral cortical projections in monkeys (see Barbas et al., BMC Neuroscience 2005).

Agreed. We have included citations to this literature in the text.

(“Results” section, “Common organizational patterns of connectivity modes” subsection, Paragraph #1):

> “Homotopic connections stand out, indicating that homologous cortical regions in left and right hemispheres are consistently similar to each other no matter the biological feature (Fig. S1b; (Barbas et al 2005, Goulas et al 2017))”

Barbas, H., Hilgetag, C. C., Saha, S., Dermon, C. R., & Suski, J. L. (2005). Parallel organization of contralateral and ipsilateral prefrontal cortical projections in the rhesus monkey. *BMC neuroscience*, *6*(1), 1-17.

Goulas, A., Uylings, H. B., & Hilgetag, C. C. (2017). Principles of ipsilateral and contralateral cortico-cortical connectivity in the mouse. *Brain Structure and Function*, *222*, 1281-1295.

6) Page 2, Results Common organizational principles of connectivity modes, paragraph 2: "Furthermore, similarity between brain regions decreases as both Euclidean and geodesic distance between brain regions increases (Fig. 1b; Fig. S2), consistent with the notion that proximal neural elements are more similar to one another [47, 60, 67, 120, 137]" and later in Page 3, "These differences are greater than in a population of degree- and edge length-preserving surrogate structural connectomes, indicating that the effect is specifically due to wiring rather than spatial proximity [21]" and the section "Structural and geometric features of connectivity modes" the authors could place the current findings and their interpretation within the framework of prior work after reading and citing as needed relevant studies that have made similar points by e.g., Garcia-Cabezas et al., Brain Structure and Function 2019; Aparicio-Rodríguez and García-Cabezas, Cerebral Cortex 2023; Beul et al., Brain Structure and Function 2015; Beul et al., Scientific Reports 2017; Hilgetag et al., Network Neuroscience 2019.

We now present and interpret the results in light of neuroanatomical work in model organisms. We emphasize how the present study complements the neuroanatomical literature by expanding these

principles of structural connectedness to multiple other biological phenotypes (i.e. not just cytoarchitecture but also genes, receptors, metabolism, and dynamics) at the level of the human whole-cortex.

("Results" section, "Common organizational patterns of connectivity modes" subsection, Paragraph #2–3):

> "Visually, each connectivity mode demonstrates non-random network organization, which we explore in subsequent sections. Furthermore, similarity between brain regions decreases as both Euclidean and geodesic distance between brain regions increases (Fig. 1b; Fig. S2), consistent with the notion that proximal neural elements are more similar to one another (Hilgetag et al 2019, Garcia-Cabezas et al 2019, Richiardi et al 2015, Fornito et al 2019, Hansen et al 2022, Shafiei et al 2020). However, there is variability in how feature similarity decreases with distance. For instance, dynamic modes demonstrate stronger exponential relationships whereas molecular modes demonstrate either weak exponential or linear (in the case of laminar similarity) fits.
>
> We next sought to relate each connectivity mode to the brain's underlying structural architecture. We constructed a weighted structural connectome using diffusion-weighted MRI data from the Human Connectome Project; this network represents whether, and how much, two brain regions are connected by white matter streamlines. We find that, across all seven connectivity modes, cortical regions that are physically connected by white matter show greater feature similarity than those that are not connected, suggesting that biologically similar neuronal populations are in direct communication (Fig. 1c). These differences are greater than in a population of degree- and edge length-preserving surrogate structural connectomes, indicating that the effect is specifically due to wiring rather than spatial proximity (Betzel et al 2018). Notably, neuroanatomical studies in model organisms have found that cytoarchitectonic similarity predicts neuronal projections better than distance (Beul et al 2015, Beul et al 2017, Aparicio-Rodriguez et al 2023); we expand on this by showing that all connectivity modes demonstrate greater similarity for structurally connected brain regions in the human. Finally, for the subset of edges with a structural connection, we find a correlation between the strength of the structural connection and each connectivity mode's edge weight (Fig. 1d) (Honey et al 2009). Altogether, we find that connectivity modes demonstrate commonalities that respect distance, neuroanatomy, and anatomical connectivity, regardless of imaging modality or biological mechanism."

("Results" section, "Structural and geometric features of connectivity modes" subsection, Paragraph #2):

> "Spatial proximity influences inter-regional similarity, such that proximal regions tend to share similar biological and physiological features (Fig. 2b, (Stiso et al 2018, Betzel et al 2018, Aparicio-Rodriguez et al 2023)."

("Results" section, "Structural and geometric features of connectivity modes" subsection, Paragraph #3):

> "This lets us determine which connectivity modes demonstrate the greatest coupling between high inter-regional feature similarity and structural connectivity. Previous work has found a close correspondence between cytoarchitecture and neuronal projections (Garcia-Cabezas et al 2019, Barbas et al 2015); our findings suggest a possible genetic

and neuroreceptor mechanism underlying this relationship. The primacy of molecular connectivity modes is a finding that returns in the next analysis and when we compare connectivity modes to disease pathology (*Connectivity modes and disease-specific abnormal cortical thickness*)."

("Results" section, "Structural and geometric features of connectivity modes" subsection, Paragraph #5):

"We find that edges in the brain's topological rich club regime are particularly dominated by molecular features (e.g. laminar similarity, correlated gene expression, and receptor similarity) (Beul et al 2015). Haemodynamic and electrophysiological connectivity are especially weak for links between high-degree regions, and temporal similarity is unstable. Metabolic connectivity is an additional connectivity mode that demonstrates significantly increased edge strength for links between high-degree regions, suggesting that energy consumption is synchronized between structural hubs (Fulcher et al 2016, Arnatkeviciute et al 2021, Liang et al 2013, Vaishnavi et al 2010). Collectively, these findings indicate that the rich club may reflect coordinated patterns of inter-regional microscale similarity across multiple molecular features. On the other hand, the rich club is not characterized by similar neural dynamics, possibly related to the functional flexibility of these regions (Griffa et al 2018)."

Finally we have added a new paragraph in the Discussion about the importance of neuroanatomical work (see also our response to Reviewer #1 Comment #4 and later on in Reviewer #2 Comment #11).

("Discussion" section, Paragraph #10):

"Throughout this report, we have illustrated how cortical connectivity can be extended from neural wiring to many complementary perspectives of inter-regional relationships. We focus on densely sampled data across the whole-cortex to make general claims about patterns of cortical organization. Most of the data employed are derived from *in vivo* neuroimaging in relatively large samples of healthy adult humans. However, these questions about multiscale cortical organization can—and have, for decades—be asked with more anatomical specificity using methods such as cell staining and tract-tracing in small samples of *ex-vivo* brains, generally from model organisms (Zilles et al 1988, Barbas 1986, Garcia-Cabezas et al 2019). Such studies have demonstrated that neurons make projection patterns that are tightly linked to the cellular architecture of the cortex, including the laminar differentiation of the source and target of a neural projection (Barbas & Rempel-Clower 1997) (e.g. the Structural Model, reviewed in Garcia-Cabezas et al 2019). Intertwined with laminar differentiation and tract-tracing projection patterns is also the phylogenetic age of the cortical region (Garcia-Cabezas et al 2022), markers of plasticity and stability (Garcia-Cabezas et al 2017), and likely also receptor architecture (Palomero et al 2019, Froudist-Walsh et al 2023). Furthermore, the field of developmental biology presents fundamental organizational principles for how the brain develops its spatial organization and topology (Nieuwenhuys 2017). Large-scale neuroimaging connectomic studies complement biological and neuroanatomical studies by extending predictions to the scale of the whole human cortex and across many more brain phenotypes. For example we confirm that laminar similarity is related to connectivity and the brain's rich club (Beul et al 2015), but extend this to gene expression, receptor

architecture, and metabolism. The synergy between neuroanatomical and imaging fields is necessary to fully capture inter-regional relationships across multiple layers of description."

7) In Results, Cross-modal hubs, towards the end of the section (page 8) the authors state "We find that transmodal regions such as the supramarginal gyrus, superior parietal cortex, precuneus, and dorsolateral prefrontal cortex are most consistently similar to other brain regions across all connectivity modes (Fig. 3d, right). Interestingly, these transmodal regions are commonly thought of as structural hubs but here we show that they are central at multiple levels of organization. Why are some brain regions highly similar to many other regions across multiple spatial scales and biological mechanisms? We hypothesized that cross-modal hubs are more cognitively flexible and able to support higher order, evolutionarily-advanced cognitive processes. We therefore correlated cross-modal hubness with a map of evolutionary cortical expansion [68]". It would be beneficial to readers if the authors made a connection here with previous studies that have shown that these areas are similar in structure "eulaminate" regions that have significantly expanded in evolution in primates, especially humans (see Garcia-Cabezas et al., Brain Structure and Function 2022).

We thank the Reviewer for this insight as we had not previously made the link between the cross-modal hubs and more differentiated cortex. We find cross-modal hubs in brain regions that are evolutionarily larger. Previous work from Garcia-Cabezas et al has shown that these regions also demonstrate greater laminar differentiation, likely develop after regions with poorer laminar differentiation, and have greater neuron density (Garcia-Cabezas et al 2022, Garcia-Cabezas et al 2019). We now interpret our findings in light of this neuroanatomical work. We leave this interpretation in the Discussion rather than in the Results because we do not empirically test the hypothesis that cross-modal hubs exist in evolutionarily more differentiated cortex. Note that there are also some changes related to the Reviewer's next comment (Reviewer #2 Comment #8).

("Discussion" section, Paragraph #6):

> "In an effort to understand which cortical regions are consistently central across many levels of description, we identify a set of cross-modal hubs. Brain hubs are conventionally defined as regions with a relatively large number of structural connections, but this definition ignores the multiscale character of brain networks. Indeed, we find that hub profiles are not redundant across biological mechanisms. Instead, we identify a subset of cortical regions that are uniquely similar across multiple levels of description. These cross-modal hubs exist in the dorsal precuneus, supramarginal gyrus, and dorsolateral prefrontal cortex: association regions that expand in surface area and develop into more differentiated eulaminate (6-layer) cortex during evolution (Hill et al 2010, Xu et al 2020,Garcia-Cabezas et al 2019). This suggests that phylogenetic structural modifications—including increased cellular complexity and density (Garcia-Cabezas et al 2022)—may support integration of information across multiple biological scales, resulting in higher-level cognition including language, planning and complex executive functions. Interestingly, these regions are distinct from the anatomically central (e.g. limbic) regions that were previously hypothesized to be integrative general-domain hubs, based on multiple measures of centrality calculated on the structural connectome (Zhang et al 2020). Future work should investigate more deeply how structural centrality is aligned with biological feature similarity. Altogether, cross-modal hubs open a new perspective on hub function: instead of being rooted only in high structural connectivity, hubs can be

> classified according to their participation in different biological systems (Arnatkeviciute et al 2021)."

García-Cabezas, M. Á., & Zikopoulos, B. (2019). Evolution, development, and organization of the cortical connectome. *PLoS biology*, *17*(5), e3000259.

García-Cabezas, M. Á., Hacker, J. L., & Zikopoulos, B. (2022). Homology of neocortical areas in rats and primates based on cortical type analysis: an update of the Hypothesis on the Dual Origin of the Neocortex. *Brain Structure and Function*, 1-25.

8) The statement "transmodal regions are commonly thought of as structural hubs but here we show that they are central at multiple levels of organization" could be clarified further. Do the authors imply that all connectomes, including the structural, identify key hub areas that are more or less similar (contain the same areas) or not? Or is it that hubs across modalities tend to contain similar types of areas? Moreover, other studies (e.g., Zhang J, Scholtens LH, et al., Cerebral Cortex 2020) showed that highly anatomically central areas may function as "high-level connectors," integrating already highly integrated information across modules. These results are consistent with a high-level, domain-general limbic workspace, integrated by highly anatomically central cortical areas. How does that fit to the current findings for each distinct connectivity mode tested?

We apologize for being unclear. In the section about hubs, we find that hub profiles (quantified as weighted degree and shown in Figure 3b) are very diverse. This brings to light the value in studying communication hubs from perspectives other than structural connectivity (where hubs are defined by the number of connections a region makes, i.e. degree).

Given this diversity and the possibility of studying hubness from multiple perspectives, which regions show greater weighted degree across all seven connectivity modes? We quantify this simply as the median weighted degree across all weighted degree profiles. The regions that are consistently hubs across multiple connectivity modes exist in regions that have previously been shown to be structural hubs (in terms of structural connectivity (van den Heuvel & Sporns 2013)), are phylogenetically expanded in terms of surface area (Figure 3d), and as the Reviewer points out in the comment above, are found in eulaminate cortex. These regions include the supramarginal gyrus, superior parietal cortex, and dorsolateral prefrontal cortex. It makes sense that the identified cross-modal hubs are of similar "type" (that is, transmodal, phylogenetically expanded, eulaminate) because cross-modal hubness is calculated based on the similarity between brain regions (in terms of gene expression, receptor fingerprints, cytoarchitecture, metabolism, neural dynamics, etc).

In other words, we intended both points that the Reviewer mentions: (1) the specific regions identified as cross-modal hubs are called cross-modal hubs because they demonstrate larger weighted degree across multiple connectivity modes; and (2) therefore, by definition, these cross-modal hubs are more similar to other brain regions across multiple phenotypes (genes, receptors, function, etc).

We have modified the text to clarify this point ("Results" section, "Cross-modal hubs" subsection, Paragraph #4):

> "Finally, we focus on the cortical regions: given the spatial diversity of hub profiles (Figure 3b), are there regions that consistently show relatively high weighted degree—that is, are consistently similar to other brain regions—across multiple connectivity modes? We

quantify cross-modal hubness as the median hubness across connectivity modes (i.e. the median across brain plots shown in Figure 3b). We find that transmodal eulaminate regions such as the supramarginal gyrus, superior parietal cortex, dorsal precuneus, and dorsolateral prefrontal cortex are most consistently similar to other cortical regions across seven biological phenotypes, from molecular composition to neural dynamics (Figure 3d, right). Interestingly, the regions identified as cross-modal hubs are commonly thought of as hubs in the structural connectome; we find they also demonstrate large feature similarity across multiple levels of organization."

Zhang et al 2020 propose that anatomically central regions (i.e. limbic regions) integrate information across multiple modalities. We do not find that anatomically central regions are hubs across multiple modalities, in fact the median weighted degree calculated in Figure 3d is generally low in anatomically central regions. The discrepancy in these findings is likely because in our study, we are looking at multiple perspectives of "connectivity" from different data modalities, whereas Zhang et al are studying multiple perspectives of centrality defined on the structural connectome alone. In other words, Zhang et al find that limbic regions are central in terms of communication on the structural backbone, and we find that cross-modal hubs are central in terms of multiscale biological mechanisms. An interesting future direction would be to combine these two perspectives, e.g. how does hubness across connectivity modes align with centrality measures from the structural connectome?

We synthesize these ideas in the Discussion ("Discussion" section, Paragraph #6):

> "In an effort to understand which brain regions are consistently central across many levels of description, we identify a set of cross-modal hubs. Brain hubs are conventionally defined as regions with a relatively large number of structural connections, but this definition ignores the multiscale character of brain networks. Indeed, we find that hub profiles are not redundant across biological mechanisms. Instead, we identify a subset of brain regions that are uniquely similar across multiple levels of description. These cross-modal hubs exist in the dorsal precuneus, supramarginal gyrus, and dorsolateral prefrontal cortex: association regions that expand in surface area and develop into more differentiated eulaminate (6-layer) cortex during evolution (Hill et al 2010, Xu et al 2020,Garcia-Cabezas et al 2019). This suggests that phylogenetic structural modifications—including increased cellular complexity and density (Garcia-Cabezas et al 2022)—may support integration of information across multiple biological scales, resulting in higher-level cognition including language, planning and complex executive functions. Interestingly, these regions are distinct from the anatomically central (e.g. limbic) regions that were previously hypothesized to be integrative general-domain hubs, based on multiple measures of centrality calculated on the structural connectome (Zhang et al 2020). Future work should investigate more deeply how structural centrality is aligned with biological feature similarity. Altogether, cross-modal hubs open a new perspective on hub function: instead of being rooted only in high structural connectivity, hubs can be classified according to their participation in different biological systems (Arnatkeviciute et al 2021)."

Zhang, J., Scholtens, L. H., Wei, Y., Van Den Heuvel, M. P., Chanes, L., & Barrett, L. F. (2020). Topography impacts topology: anatomically central areas exhibit a "high-level connector" profile in the human cortex. *Cerebral Cortex*, *30*(3), 1357-1365.

Van den Heuvel, M. P., & Sporns, O. (2013). Network hubs in the human brain. *Trends in cognitive sciences*, *17*(12), 683-696.

9) I would appreciate more information about the relationship of exposure and negative edge strength in the Results "Connectivity modes shape disease vulnerability". Does a negative edge strength play any role in exposure calculation? Could a negative edge strength indicate resilience?

In the original analysis, a source node's "disease exposure" was defined as the average regional abnormality (as defined by the specific spatial cortical disorder profile) for all regions that are positively connected (i.e. have a positive edge weight) with a source node, weighted by the magnitude of the connection (i.e. the edge weight). That is, negative edges did not play any role in exposure calculation. We originally selected only positive edges because it serves as a natural thresholding of the network and makes disease exposure easier to interpret.

Nonetheless, we agree with the Reviewer that it would be interesting to know how negatively-connected edges are influencing the exposure and ultimately the alignment between predicted and empirical spatial abnormal cortical thickness patterns. To test this, we ran two analyses: (1) we consider exclusively negative edges, and (2) we consider all (positive and negative) edges. We have updated the Results and Discussion, and have added a new Supplementary Figure (Figure S5). We detail the methodology, findings, and updated text below.

(1) Considering exclusively negative edges:
We threshold each network such that only negative edges remain, then take the absolute value of the network to ensure mean abnormality remains positive. In this case, a source node's disease exposure is the average regional abnormality for all regions that are negatively connected with a source node, weighted by the magnitude of the connection.

We find that nearly all correlations between disease exposure and empirical abnormal cortical thickness are negative: regions with high abnormality are negatively connected to regions with low abnormality and vice versa. By comparing these correlations with the original correlation coefficients when using only positive edges, we find that positive and negative edges have opposing effects on disease exposure (Spearman r=-0.52). This may indicate some resilience effect: for regions with high abnormality, the spread of pathology may be attenuated by negative edges; likewise for regions with low abnormality, they may be protected from high abnormality regions by negative edges (which represents dissimilar or anticorrelated local features).

(2) Considering all edges:
Here we define a source region's exposure as the mean abnormality of all other regions in the brain, weighted by edge strength. Positive and negative signs are unchanged such that when calculating the weighted average, positively-connected regions increase exposure and negatively-connected regions decrease exposure.

Here we find very similar results to the original (positive edge only) analysis (Figure 4) although most correlation coefficients are now slightly smaller. This suggests that the strongest connections (i.e. the positively connected edges) have the greatest influence in predicting disease exposure and the empirical cortical disorder profile.

Figure S5:

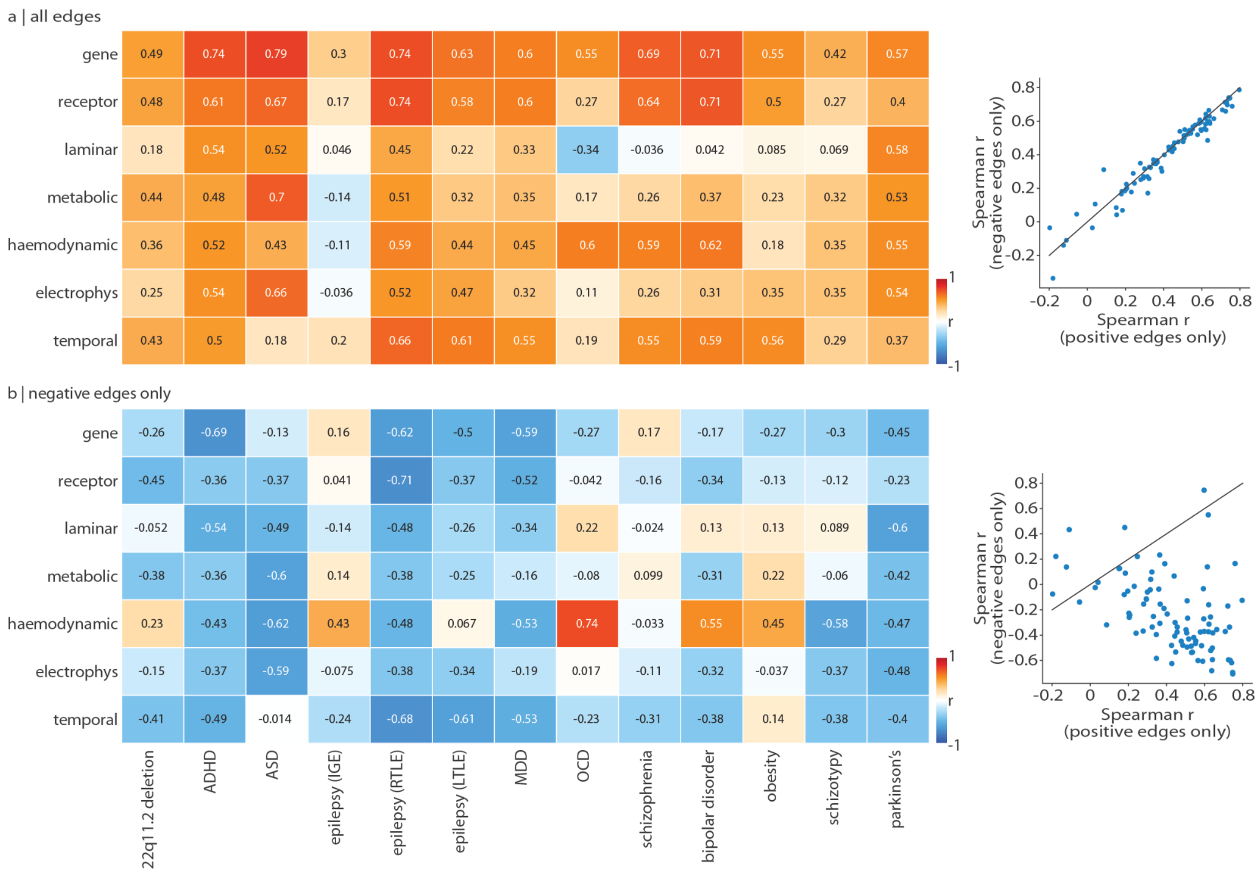

"Figure S5. Investigating the influence of negative edges in disease exposure | We repeat the procedure in Fig. 4 but instead of retaining only positive edges, we retain (a) all edges, and (b) only negative edges. Scatter plots compare the original (positive edges only; x-axis) and updated (y-axis) Spearman's *r* correlation coefficients, with the identity line shown."

("Results" section, "Connectivity modes and disease-specific abnormal cortical thickness" subsection, Paragraph #3):

"We find that correlated gene expression and receptor similarity most consistently amplify the exposure of pathology in a manner that closely resembles the structural cortical profile of the disease. Interestingly, when we repeat the analysis using only negative edges (c_{ij} if $c_{ij} < 0$), we find opposite results: cortical regions with high abnormality are negatively connected (that is, are dissimilar to) regions with low abnormality (Figure S5b). This suggests that dissimilarity may attenuate disease spread. By repeating the analysis using weighted structural connectivity (in which case we only consider structurally-connected regions) and Euclidean distance between brain regions (in which case we always consider the full network), we are able to uncover cases where feature similarity amplifies disease exposure more than structure or distance alone (Fig. 4c). Abnormal cortical thickness patterns of psychiatric disorders in particular (e.g. MDD, schizophrenia, bipolar disorder, OCD) are better explained by correlated gene expression and receptor similarity than structure or distance. This integrative analysis makes it possible to hone in on the imaging modalities and biological mechanisms that might most

reflect cortical pathology in a disease-general manner. Furthermore, it demonstrates the value in employing feature similarity as a network rather than limiting network models to the structural connectome.”

(“Discussion” section, Paragraph #8):

“Second, molecular feature similarity---particularly correlated gene expression and receptor similarity---best explains the spatial patterning of multiple cortical disease abnormalities. Recent work has explored the idea that multiple pathologies spread trans-synaptically, including misfolded proteins, aberrant neurodevelopmental signals, and excitotoxic electrical discharge, resulting in patterns of pathology that reflect the underlying structural architecture of the brain (Zheng et al 2019, Shafiei et al 2019). Here we consider the possibility that shared vulnerability to disease arises not just from structural connectivity but also from multiscale biological attributes (Hansen et al 2022). We use changes in cortical thickness as the marker of potential pathology and find that when disease exposure is informed by transcriptional and receptor similarity, we can reproduce the cortical profile of multiple diseases (r > 0.5 for most). We also find evidence that molecular dissimilarity may serve as a mitigating factor of pathological spread (Figure S5), although more work is necessary to determine the link between regional dissimilarity and pathology. The consistent primacy of molecular connectivity modes demonstrates that mapping cortical connectivity from the perspective of the underlying biology---gene transcription, receptor density, cellular composition---is just as, if not more, informative than oft-studied dynamical modes such as haemodynamic connectivity. This analysis can be extended in future work by studying the connectivity modes of patient populations, and will hopefully motivate future causal work linking molecular mechanisms to the spreading of pathological markers in the brain.”

10) It would be interesting to further examine how the findings from the current study fit/compare with relevant prior work with supporting or contrasting previous findings. For example, an elegant study by Goulas et al., PNAS 2021 uncovered a receptor-based natural axis of the human cerebral cortex, which also parallels laminar architecture and showed that on the sensory extreme of cortical gradients of laminar elaboration there is less diversity of receptors, more inhibition, less excitation, more ionotropic and fewer metabotropic receptors. On the association extreme of cortical gradients of laminar elaboration there is more diversity of receptors, less inhibition, more excitation, fewer ionotropic and more metabotropic receptors. I believe it would be interesting to add a comment about these observations and compare associations made in the two studies, especially given the following statement in page 9 "Furthermore, we find that brain gradients do not all follow a uniform sensory association axis [73, 96, 149], rather, the first principal component of each connectivity mode varies considerably".

We thank the Reviewer for this suggestion and apologize for neglecting Goulas et al 2021 PNAS as it is certainly very relevant to the section about gradient organization. Goulas et al 2021 is a good example of how gradients can help us understand brain organization as well as how microscale mechanisms may gives rise to brain function - in this case, gradients of excitation/inhibition mediated by receptor density resulting in an alignment with the functional sensory-association gradient. We likely get different gradients because we are calculating the first gradient of the region x region connectivity mode (i.e. similarity matrix) rather than the region x receptor density matrix. These gradients are interpreted differently: in our case, we are looking at organizational principles of “connectivity” (that is, similarity) between regions

according to receptor density; in Goulas et al 2021, they are looking at an axis of variation of receptor density.

We have added a new paragraph in the Discussion about gradients/modules, how they can be used to infer brain organization (e.g. as shown by Goulas et al 2021), and to clarify that our gradients are derived from similarity networks rather than from the underlying data (see also our response to Reviewer #3 Comment #6).

(“Discussion” section, Paragraph #9):

> “Lastly, we examine the gradient and modular organization of connectivity modes. Low-dimensional topographical representations of brain features, whether spatially continuous (gradients) or discrete (modules), present insight on how different levels of cortical organization are aligned with one another. For example, graded changes in the proportion of neural projections originating from upper versus lower laminar layers has been related to gradients of pyramidal neuron soma size, number of synaptic boutons, number of vesicles, amount of neurotransmitter release, and firing rate, providing a comprehensive explanation for the laminar origin of cortico-cortical connections (Goulas et al 2018). Likewise, Goulas et al 2021 showed that gradients of receptor density reflect excitatory/inhibitory and ionotropic/metabotropic ratios and follow a sensory-association functional hierarchy. Here we view local molecular and dynamic features from the perspective of a network to study the organization of cortical “connectivity”, rather than axes of variation of the underlying data (Goulas et al 2021, Hansen et al 2022, Burt et al 2018). This approach lets us apply not only gradient decomposition but also community detection - rarely used in brain imaging outside of structural and haemodynamic connectivity - to the networks. We find that connectivity modes have unique gradient and modular decomposition which means it is not sufficient to assume a single spatial organization for the cortex. Interestingly, previous work has found that fMRI-derived functional communities can themselves be diverse: they fluctuate over time, during tasks, and throughout hormonal cycles (Bassett et al 2011, Bassett et al 2013, Mueller et al 2021). These temporary changes in network organization may reflect the diverse modular organization of underlying molecular mechanisms. The biological origins of the diversity in spatial gradients and modules will be an important direction for future research (Huntenburg et al 2018, Bernhardt et al 2022).”

11) In addition, several other studies, reviewed in Garcia-Cabezas et al., Brain Structure and Function 2019 have linked structure of the cortex with presence/absence, strength, and laminar pattern of connections and function in terms of feedforward-feedback-lateral processing in the cortex. Again, it would be interesting to add a comment about how the findings from the current study fit/compare with these data.

We now contextualize the findings with respect to studies linking laminar architecture with neural connectivity. We have added a new paragraph to the Introduction to prime readers on this subject (see also our response to Reviewer #2 Comments #2 & #3; “Introduction” section, Paragraph #2):

> “However, the graph representation of the structural connectome, in which regional nodes are identical, does not account for the molecular and physiological heterogeneity that exists in the brain. An emerging representation of connectivity is feature similarity: if two brain regions exhibit similar biological features, we might expect them to be related to

one another and engaged in common function (Zilles et al 2015, Anderson et al 2018, Paquola et al 2019, Voigt et al 2022, Horwitz et al 1984). Neuroanatomical tract-tracing studies in non-human primates have extensively shown that biological feature similarity is fundamental to brain organization (Barbas 1986, Garcia-Cabezas et al 2019). These pioneering studies demonstrated that neuronal projection patterns can be predicted based on the laminar differentiation of the source and target regions (Barbas & Rempel-Clower 1997), and has been extended to human prefrontal cortex and other model organisms (Zikopoulos et al 2018, Goulas et al 2019, Beul et al 2015). Furthermore, local differences in laminar architecture follow a gradient of receptor density (Goulas et al 2021, Hansen et al 2022), and synaptic plasticity (Garcia-Cabezas et al 2017), indicating an alignment between multiple local features and connectivity. However, these studies are currently limited to qualitative measurements of cytoarchitectonic similarity, small subsets of brain regions, model organisms, and to a single perspective of molecular make-up."

We have also added a new paragraph to the Discussion, also shown in response to Reviewer #1 Comment #4 and Reviewer #2 Comment #6 ("Discussion" section, Paragraph #10):

"Throughout this report, we have illustrated how cortical connectivity can be extended from neural wiring to many complementary perspectives of inter-regional relationships. We focus on densely sampled data across the whole-cortex to make general claims about patterns of cortical organization. Most of the data employed are derived from *in vivo* neuroimaging in relatively large samples of healthy adult humans. However, these questions about multiscale cortical organization can---and have, for decades---be asked with more anatomical specificity using methods such as cell staining and tract-tracing in small samples of *ex-vivo* brains, generally from model organisms (Zilles et al 1988, Barbas 1986, Garcia-Cabezas et al 2019). Such studies have demonstrated that neurons make projection patterns that are tightly linked to the cellular architecture of the cortex, including the laminar differentiation of the source and target of a neural projection (Barbas & Rempel-Clower 1997) (e.g. the Structural Model, reviewed in Garcia-Cabezas et al 2019). Intertwined with laminar differentiation and tract-tracing projection patterns is also the phylogenetic age of the cortical region (Garcia-Cabezas et al 2022), markers of plasticity and stability (Garcia-Cabezas et al 2017), and likely also receptor architecture (Palomero et al 2019, Froudist-Walsh et al 2023). Furthermore, the field of developmental biology presents fundamental organizational principles for how the brain develops its spatial organization and topology (Nieuwenhuys 2017). Large-scale neuroimaging connectomic studies complement biological and neuroanatomical studies by extending predictions to the scale of the whole human cortex and across many more brain phenotypes. For example we confirm that laminar similarity is related to connectivity and the brain's rich club (Beul et al 2015), but extend this to gene expression, receptor architecture, and metabolism. The synergy between neuroanatomical and imaging fields is necessary to fully capture inter-regional relationships across multiple layers of description."

12) In "Connectivity modes shape disease vulnerability" and associated Fig. 4, the authors show how we can use this approach to hone in the imaging modalities and biological mechanisms that might most reflect cortical pathology. Later in the presentation of the results of the fusion of connectivity modes, the authors state that the fused network maps onto intrinsic networks and cytoarchitectonic classes better than any individual network, demonstrating how large-scale

phenomena can emerge from a confluence of multiple determinants. Several diseases like ASD could be described as complex, large-scale phenomena, therefore it would be interesting to see whether the fused network could also map disease vulnerability better than any individual network and add this to Fig. 4.

We thank the Reviewer for this suggestion and agree that it would be interesting to know whether the fused network can reproduce empirical abnormal cortical thickness patterns better than individual connectivity modes. We ran the analysis in Figure 4 using the fused network and found that the fused network's performance is average. This may reflect that by fusing connectivity modes, the benefits of molecular connectivity modes are washed out by the dynamic modes, which perform less well. We've included a new figure in the Supplement showing the original Figure 4 alongside the results from the fused network (last row). We don't add the extra row to the original Figure 4 because the introduction of the fused network doesn't appear until after the section about disease vulnerability.

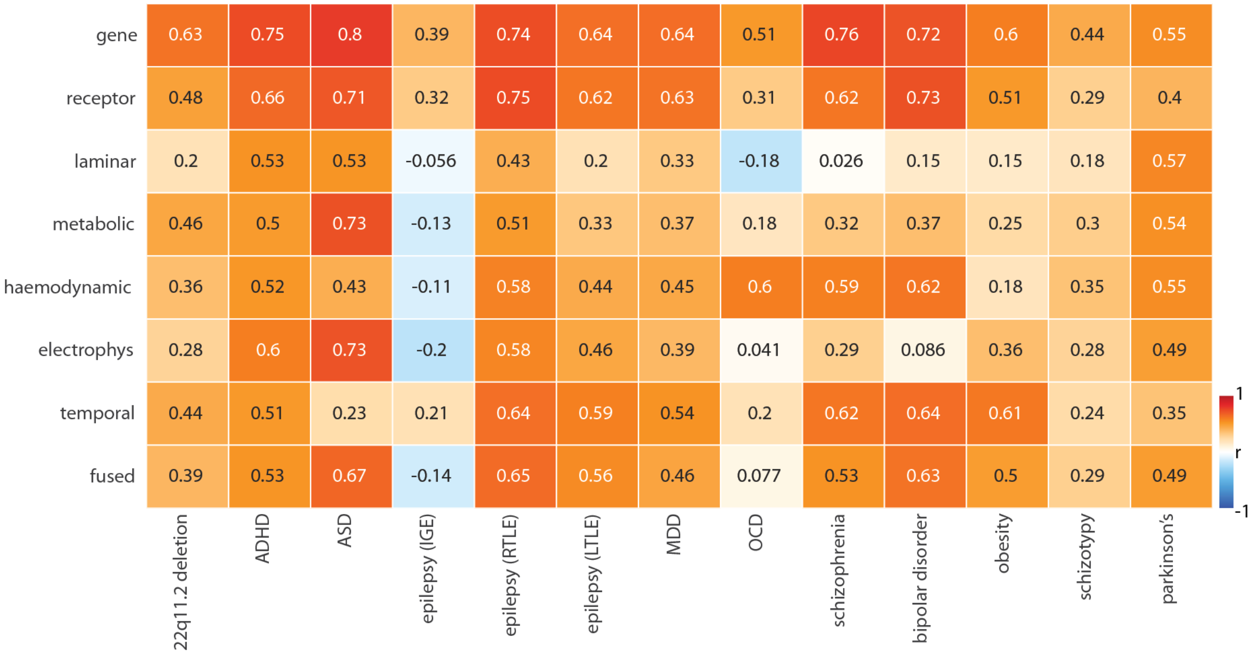

"Figure S6. Contributions of connectivity modes including the fused network to disease vulnerability | We repeat the procedure in Fig. 4 using the fused network from the analysis in Fig. 6 (bottom row). The first seven rows of the heatmap, corresponding to the seven connectivity modes, are identical to those shown in Fig. 4 and are repeated to facilitate comparison with the fused network. We find that the fused network performs average, which may reflect that by fusing connectivity modes, the benefits of molecular connectivity modes in predicting disease patterns are washed out by the dynamic modes which perform less well."

13) The structural connectivity dataset used may over-represent long-range cortical connections, which tend to be more common between eulaminate areas. The authors clarify that each dataset has its own limitations however, it seems that actual connectivity between areas (based on the structural connectivity dataset) weighs more during integration of multiple modes and within each mode for hub identification. The authors should discuss how this could affect cross-modal hub identification and other relevant findings.

We agree with the Reviewer that many results depend on the group-consensus structural connectome, and apologize for forgetting to include it as an important limitation of the study. The structural connectome comes into play in multiple places:

- When calculating edge weight for connected versus not connected regions (Figure 1c and Figure 6d)
- When calculating the correlation between structural connectivity and connectivity modes (Figure 1d and Figure 6d)
- When comparing rank-transformed edge weights for structurally-connected edges (Figure 2b)
- When estimating the rich club regime (Figure 2c)
- When calculating median edge rank for edges between regions with structural degree >= *k* (Figure 2d)
- When calculating disease exposure on the structural network alone as a benchmark (Figure 4c)

Note that the structural connectome is not used when calculating hubness nor the cross-modal hub maps. These maps (Figure 3) are calculated on the connectivity mode alone (i.e. the networks shown in Figure 1a).

The structural connectomes employed in this analysis were processed using a procedure that explicitly limits the over-representation of long-range connections (by seeding at the grey matter-white matter boundary rather than in the white matter; (Smith et al 2015; Jeurissen et al 2017)). Therefore, in our case, the concern is not so much an over-representation of long-range connections but instead the general presence of false positives and false negatives in diffusion tractography. We attempted to mitigate this issue by constructing a length-dependent group-consensus network where we keep frequently-occuring edges within specific length bins (across participants), thereby only considering edges that are consistently present across people.

We have added a point to the Discussion emphasizing the limitations of the structural network ("Discussion" section, Paragraph #11):

> "Second, each connectivity matrix is dependent on the quality of the imaging modality, and each imaging method operates at a unique spatial and temporal resolution. Results may therefore be influenced by differences in how the data are acquired. In addition to this, the group-consensus structural network that was used throughout the analyses (in particular Figure 1, 2, 4 and 6) was reconstructed from diffusion spectrum imaging and tractography, which is prone to false-positives and false-negatives (Maier-Hein et al 2017, Zalesky et al 2016)."

Jeurissen, B., Descoteaux, M., Mori, S., & Leemans, A. (2019). Diffusion MRI fiber tractography of the brain. *NMR in Biomedicine*, *32*(4), e3785.

Smith, R. E., Tournier, J. D., Calamante, F., & Connelly, A. (2015). SIFT2: Enabling dense quantitative assessment of brain white matter connectivity using streamlines tractography. *Neuroimage*, *119*, 338-351.

Maier-Hein, K. H., Neher, P. F., Houde, J. C., Côté, M. A., Garyfallidis, E., Zhong, J., ... & Descoteaux, M. (2017). The challenge of mapping the human connectome based on diffusion tractography. *Nature communications*, *8*(1), 1349.

Zalesky, A., Fornito, A., Cocchi, L., Gollo, L. L., van den Heuvel, M. P., & Breakspear, M. (2016). Connectome sensitivity or specificity: which is more important?. *Neuroimage*, *142*, 407-420.

14) The main limitations arise from compiling data for each modality across age, sex, and other key factors, like handedness (not mentioned in this article), limitations of each dataset used e.g., non-specific binding in PET datasets, and challenges when comparing inherently different types of data. Most were addressed adequately by the authors. As the authors stated, all limitations can be addressed in future in-depth studies, as new datasets become available for each feature used to determine these inter-regional similarity estimates however, I would clarify that they can also be addressed by concurrently using multiple datasets for each type of similarity estimate that are currently available. The authors addressed transmodal comparison challenges through an elegant study design that included alternative parcellation schemes, rank-transformations, and normalization of data. Therefore, despite the limitations, the findings are novel, highly significant, and the manuscript is poised to be an outstanding contribution to our field and of general interest.

We have added this paragraph from the Reviewer's preamble as a final point because we have updated the limitations to include handedness as well as the Reviewer's point about concurrently using multiple datasets to test multiple similarity networks.

("Discussion" section, Paragraph #11):

> "The present work should be considered alongside some methodological considerations. First, the results are only representative of the seven included connectivity modes; future work should replicate the findings in similar connectivity modes derived from external datasets, as well as extend this work into additional forms of connectivity. …
> … Fourth, connectivity modes are compiled across different individuals of varying ages, sex ratios, and handedness. Results are therefore limited to group-averages, and motivate future deep phenotyping studies of the brain across multiple scales and modalities."

Basilis Zikopoulos

Reviewer #3:

In this work, Hansen and colleagues explored different facets of brain connectivity, using measures of gene expression, receptor density, cellular composition, metabolism, electrophysiology, and temporal features. Specifically, they estimated a "connectivity mode" for each of these measures and investigated their common organizational principles and contributions to brain structure and geometry, also linking these measures with spatial patterns of cortical abnormality in a wide range of brain disorders.
Overall, this is a very interesting piece of work, and finds its own space in the series of works from this group related to the topic of brain connectomics. The manuscript is well written, but I'd like to ask the authors to address some points before endorsing it for publication.

1) In section "Connectivity modes shape disease vulnerability", the authors say: "We next ask how connectivity modes shape the spatial patterning of brain diseases". Although I get what the authors mean, I'm not sure that the connectivity modes can 'shape' the patterning of brain diseases, nor that this can be tested in this work, as the only data of clinical cohorts used here are measures of structural abnormality, so not directly related to the modes investigated. Also, the authors seem to suggest that structural abnormalities define the spatial patterning of brain diseases, but the spatial patterning of brain diseases is much more complex than this, and that the anatomical modifications are just part of the full picture. Some lines below, the authors use the term "cortical profile of the disease". This definition sounds more in line with what the authors probably want to indicate, although I would highlight that it's the structural profile of the disease.

We agree with the Reviewer and apologize for the unclear terminology. We now clarify that the disease profile is a structural cortical disease profile (not a general spatial patterning of disease). Furthermore we remove the causal language (e.g. “shape”, “is informed by”) in the text since this analysis is purely correlational (see also our response to the Reviewer’s next comment).

New section title: “Connectivity modes and disease-specific abnormal cortical thickness” (also in response to the Reviewer’s Comment #14).

(“Results” section, “Connectivity modes and disease-specific abnormal cortical thickness” subsection, Paragraph #1):

> “~~We next ask how connectivity modes shape the spatial patterning of brain diseases. is mediated by shared molecular vulnerability.~~ Pioneering studies in post-mortem tissue gave rise to the theory that the physicochemical composition of neurons at local brain regions results in a selective vulnerability to brain disease (Klatzo 2003). Other classical studies have shown that disease propagation in the cerebral cortex is related to microscale features such as myelination (Braak & Braak 1996). These propagation patterns have been successfully modeled at the level of the whole-cortex using the structural connectome, and often perform better when informed by local biological features such as the expression of a specific gene (Henderson et al 2019, Shafiei et al 2023). Recent findings build on this notion and posit that the course and expression of multiple brain diseases on the structural connectome is mediated by multiple forms of molecular vulnerability rather than a single molecular perturbation (Warren et al 2013, Hansen et al 2022). We therefore tested whether disease propagation patterns derived from the connectivity modes could predict abnormal cortical thickness patterns for thirteen different neurological, psychiatric, and neurodevelopmental diseases and

> disorders from the ENIGMA consortium (N=21,000 patients, N=26,000 controls) (Thompson 2020, Lariviere et al 2021, Hansen et al 2022). The disease-specific abnormal cortical thickness patterns are regional z-scored case-versus-control effect sizes, representing deviation from normative cortical thickness. We refer to these regional values as "abnormal cortical thickness" or simply "abnormality"."

("Results" section, "Connectivity modes and disease-specific abnormal cortical thickness" subsection, Paragraph #3):

> "We find that correlated gene expression and receptor similarity most consistently amplify the exposure of pathology in a manner that closely resembles the structural cortical profile of the disease."

2) In the same section, the authors say: "Finally, we correlate cortical abnormality with disease exposure to determine whether the spatial patterning of the disease is informed by a connectivity mode". I'm not quite convinced about the meaning of this sentence. What the authors are doing here is a correlation between structural alterations and connectivity modes, which means that they are checking if there is a link between the connectivity modes in the healthy brain and abnormal cortical thickness. The wording "is informed by" gives me the impression that, for example, if a correlation with the receptor mode exists, this mode is influencing the anatomical abnormality, but this is not necessarily the case and cannot be tested in this specific study.

We agree and have removed the causal language from this section.

("Results" section, "Connectivity modes and disease-specific abnormal cortical thickness" subsection, Paragraph #2):

> "Finally, we correlate abnormal cortical thickness with disease exposure to determine whether the disease demonstrates a cortical disease profile that reflects the underlying connectivity mode (Fig. 4a, right). Given a disease where greater disease exposure results in greater abnormal cortical thickness, we would expect to find a large positive correlation."

And we have expanded the discussion to include the importance of causal studies linking molecular mechanisms to pathological spreading ("Discussion" section, Paragraph #8):

> "Recent work has explored the idea that multiple pathologies spread trans-synaptically, including misfolded proteins, aberrant neurodevelopmental signals, and excitotoxic electrical discharge, resulting in patterns of pathology that reflect the underlying structural architecture of the brain (Zheng et al 2019, Shafiei et al 2020). Here we consider the possibility that shared vulnerability to disease arises not just from structural connectivity but also from multiscale biological attributes (Hansen et al 2022). We use changes in cortical thickness as the marker of potential pathology and find that when disease exposure is estimated by transcriptional and receptor similarity, we can reproduce the cortical profile of multiple diseases ($r > 0.5$ for most). We also find evidence that molecular dissimilarity may serve as a mitigating factor of pathological spread (Figure S5), although more work is necessary to determine the link between regional dissimilarity and pathology. The consistent primacy of molecular connectivity modes demonstrates that mapping cortical connectivity from the perspective of

> underlying microscale features---gene transcription, receptor density, cellular composition---is just as, if not more, informative than oft-studied dynamical modes such as haemodynamic connectivity. This analysis can be extended in future work by studying the connectivity modes of disease cohorts, and will hopefully motivate future causal work linking molecular mechanisms to the spreading of pathological markers in the brain."

3) Same section: the authors should mention in this section that what they mean by "cortical abnormality" is "abnormal cortical thickness", as the term used is too generic.

Agreed. We have updated the terminology from "cortical abnormality" to "abnormal cortical thickness" in almost all instances, including in the subsection's heading. We have added a sentence to the first paragraph of this subsection to define the terminology:

("Results" section, "Connectivity modes and disease-specific abnormal cortical thickness" subsection, Paragraph #1):

> "The disease-specific abnormal cortical thickness patterns are regional z-scored case-versus-control effect sizes, representing deviation from normative cortical thickness. We refer to these regional values as "abnormal cortical thickness" or simply "abnormality"."

4) Section "Gradients and modules of connectivity nodes" would benefit more details. For example: To give the readers a bit of context, I would provide a definition of gradients and modules and explain why they could be important to understand the connectivity modes.
5) About the modules, I would find useful for non-expert readers an explanation of what the resolution parameter is, and what it means in practical terms that the solution is stable/unstable to define and understand the network's characteristics.

We reply to both comments together because they appear in the same section. We have reworked the section on gradients and modules to be more accessible to a broader audience. This includes motivating and defining gradients and modules, the resolution parameter, and our use of "stable" and "unstable" community detection solutions.

("Results" section, "Gradients and modules of connectivity modes" subsection):

> "We next consider how each connectivity mode is intrinsically organized, both in terms of axes of variation (i.e. spatial gradients) and network modules (Hanisch et al 2022, Dear et al 2022, Margulies et al 2016, Paquola et al 2019). The principal gradient, quantified as the first principal component of a connectivity mode, is a regional quantification of how feature similarity varies across the cerebral cortex. They can be interpreted as a single-dimensional representation of the connectivity mode and will highlight the regions that are especially similar to or dissimilar from one another. We start by studying an under-appreciated element of the principal component: how much variance is explained by (i.e. how representative is) each principal gradient? We find that the prominence of the first gradient can vary substantially across connectivity modes (Figure 5a). For example, the temporal similarity gradient is especially dominant (accounting for 73.8% of variance) while the metabolic connectivity gradient is especially non-dominant (accounting for 12.7% of variance; Figure 5b). Furthermore, we find that brain gradients do not all follow

a uniform sensory-association axis (Sydnor et al 2021, Huntenburg et al 2018, Markello et al 2022), rather, the first principal component of each connectivity mode varies considerably (median absolute correlation between gradients r=0.36; Figure 5c).

An alternative perspective of intrinsic network organization comes from considering whether and how the network clusters into segregated modules (Sporns & Betzel 2016). In other words, which subsets of cortical regions are similar to one another (according to a specific connectivity mode) and are these modules consistent across connectivity modes? We apply the Louvain community detection algorithm to each connectivity mode to search for groups of regions that exhibit high within-module similarity and high between-module dissimilarity (Blondel et al 2008, Bassett et al 2013). The Louvain algorithm is unsupervised and does not require a predefined number of clusters as input; instead, the resolution parameter (γ) tunes the ease with which more communities are detected (larger γ results in more communities being identified). To get a sense of the resolution of each network (i.e. the optimal number of communities the network might naturally exhibit, if at all), we track the number of communities identified by the Louvain community detection algorithm across different values of γ (Figure 5d). We find that the community detection solution for electrophysiology is highly unstable, that is, the number of identified communities changes rapidly with small changes in γ. The most stable solution at γ=1 simply delineates the main cortical lobes which suggests that electrophysiological connectivity organization is better described as a gradient but not as distinct modules of brain regions. Haemodynamic connectivity and temporal similarity show a similar trend, where partitions of greater than approximately 5 networks become increasingly unstable. Meanwhile, correlated gene expression, laminar similarity, and receptor similarity show more stable community solutions: large changes in γ are required for the network to split itself into more communities. This suggests that molecular connectivity modes can be described from the perspective of a small number (<10) of modules. We show one possible consensus community detection solution for each network in Figure 5e, which demonstrates that the modular organization and gradient decomposition of networks tend to be closely aligned. Collectively, this shows that each connectivity mode has a unique gradient decomposition and community structure."

6) "Collectively, this shows that each connectivity mode has a unique gradient decomposition and community structure." What does it mean in practical terms? Could you further explain why this information is relevant?

Our results show that it is not sufficient to assume a single spatial organization of the cortex (for example, the unimodal-transmodal functional hierarchy or the intrinsic resting-state networks), and that each connectivity mode provides a new perspective on cortical organization. This is reflected in both gradients (smooth transitions across the cortex) and modules (discrete subsets of cortical regions). We have added a new paragraph to the Discussion to expand on the relevance and interpretation of modular and gradient organization of connectivity modes (see also our response to Reviewer #2 Comment #10).

("Discussion" section, Paragraph #9):

"Lastly, we examine the gradient and modular organization of connectivity modes. Low-dimensional topographical representations of brain features, whether spatially continuous (gradients) or discrete (modules), present insight on how different levels of

cortical organization are aligned with one another. For example, graded changes in the proportion of neural projections originating from upper versus lower laminar layers has been related to gradients of pyramidal neuron soma size, number of synaptic boutons, number of vesicles, amount of neurotransmitter release, and firing rate, providing a comprehensive explanation for the laminar origin of cortico-cortical connections (Goulas et al 2018). Likewise, Goulas et al 2021 showed that gradients of receptor density reflect excitatory/inhibitory and ionotropic/metabotropic ratios and follow a sensory-association functional hierarchy. Here we view local molecular and dynamic features from the perspective of a network to study the organization of cortical "connectivity", rather than axes of variation of the underlying data (Goulas et al 2021, Hansen et al 2022, Burt et al 2018). This approach lets us apply not only gradient decomposition but also community detection—rarely used in brain imaging outside of structural and haemodynamic connectivity—to the networks. We find that connectivity modes have unique gradient and modular decomposition which means it is not sufficient to assume a single spatial organization for the cortex. Interestingly, previous work has found that fMRI-derived functional communities can themselves be diverse: they fluctuate over time, during tasks, and throughout hormonal cycles (Bassett et al 2011, Bassett et al 2013, Mueller et al 2021). These temporary changes in network organization may reflect the diverse modular organization of underlying molecular mechanisms. The biological origins of the diversity in spatial gradients and modules will be an important direction for future research (Huntenburg et al 2018, Bernhardt et al 2022)."

7) Section "Fusing connectivity modes": Why are the edge weights so small?

SNF iteratively generates a new fusion matrix until a specified number of iterations has been reached (in our case, 20). For every network W involved in the fusion process (in our case there are 7 networks W_1, W_2, …, W_7), we calculate a P matrix and S matrix where:

- P is a normalized version of W
- S is a sparse version of P where we only keep the k most similar neighbours (using a k-nearest neighbours approach) of every region

The fusion process multiples these matrices together (in a specific way, which we won't go into detail here but the full mathematical description exists in the Methods section) and normalizes the output after every iteration. As a result, with every fusion step and every normalization step, the edge weights get smaller and smaller. Note that also by design all edge weights are positive.

This is to say that edge weights in the fused network are somewhat of a red herring. The magnitude does matter - larger values represent greater similarity - but the absolute value is a byproduct of the iterative multiplication process.

We have added a sentence in the Methods and the associated figure caption to clarify the meaning of edge weights in the fused network.

("Methods" section, "Similarity network fusion" subsection):

"After each iteration, the generated matrices are re-normalized as in Equation 8. Fusion stops when the matrices have converged or after a specified number of iterations (in our case, 20). Regions x_i and x_j will likely be neighbours in the fused network if they are neighbours in multiple similarity networks. Furthermore, if x_i and x_j are not very similar in one data type, their similarity can be expressed in another data type. Note that the edge

> weights in the final fused network is a byproduct of the iterative multiplication and normalization steps, and therefore can become very small. Greater edge magnitude represents greater similarity (no negative edges exist, by design)."

Figure 6 caption:

> "(b) The fused network. We show the matrix-representation of the network (left), the top 0.5% strongest edges of the network (bottom), and the weighted degree of each brain region (right). Note that the edge weights in the fused network is a byproduct of the iterative multiplication and normalization steps (see *Methods* for details), and therefore can become very small. Greater edge magnitude represents greater similarity (no negative edges exist, by design)."

Wang, B., Mezlini, A. M., Demir, F., Fiume, M., Tu, Z., Brudno, M., ... & Goldenberg, A. (2014). Similarity network fusion for aggregating data types on a genomic scale. *Nature methods*, *11*(3), 333-337.

8) Referring to the greater correlation between edge weight and weighted structural connectivity, the authors say: "This shows how combining inter-regional similarity across multiple scales can be used to better explain anatomical connectivity". I'm not sure about which measure can explain the other. Can this result be interpreted instead as the fact that anatomically connected regions are more likely to share similar profiles on multiple scales? So, basically, that it's the anatomical connectivity that can be used to better explain inter-region similarity across multiple scales?

We agree with the Reviewer that this interpretation can go both ways: do connections exist because local features are similar or are local features similar because connections exist? Furthermore, these two interpretations are not mutually exclusive. We have modified the sentence such that it no longer suggests causality but rather leaves the interpretation open.

("Results" section, "Fusing connectivity modes" subsection, Paragraph #2):

> "Finally, the fused network demonstrates a greater correlation between edge weight and weighted structural connectivity than any of the individual connectivity modes (r=0.53). This shows how combining inter-regional similarity across multiple scales better reflects anatomical connectivity than any single perspective of inter-regional similarity (Suarez et al 2020). This may be because regions that are similar across multiple scales are more likely to be connected, or because brain connectivity gives rise to shared biological features."

9) It would be interesting to localise in the brain map the regions/edges of the fused network, instead of having only a connectivity matrix, possibly trying to give an explanation of why regions A, B and C and their links are the core of this network.

We have added plots of strongest edges and nodes (similar to those shown in Figure 3a, b) to Figure 6. We find consistent results with the cross-modal hubs and edges shown in Figure 3d, namely connections between regions within unimodal (e.g. somatomotor, visual) networks are most dominant in the fused network. This is likely because the greatest similarity across all connectivity modes exists in regions that are older and more conserved across phylogeny. Meanwhile, the brain regions with the largest weighted degree exist in the anterior temporal cortex and superior frontal cortex. These represent brain regions with the greatest similarity to all other regions according to the fused network and can be interpreted as

regions with greater “flexibility” across connectivity modes. For example, the somatomotor cortex demonstrates low weighted degree likely because, although the somatomotor cortex is highly connected to itself (e.g. edges shown in Figure 6b), it is functionally specialized and therefore is weakly connected to the rest of the brain (e.g. brain plots shown in Figure 6b).

New Figure 6:

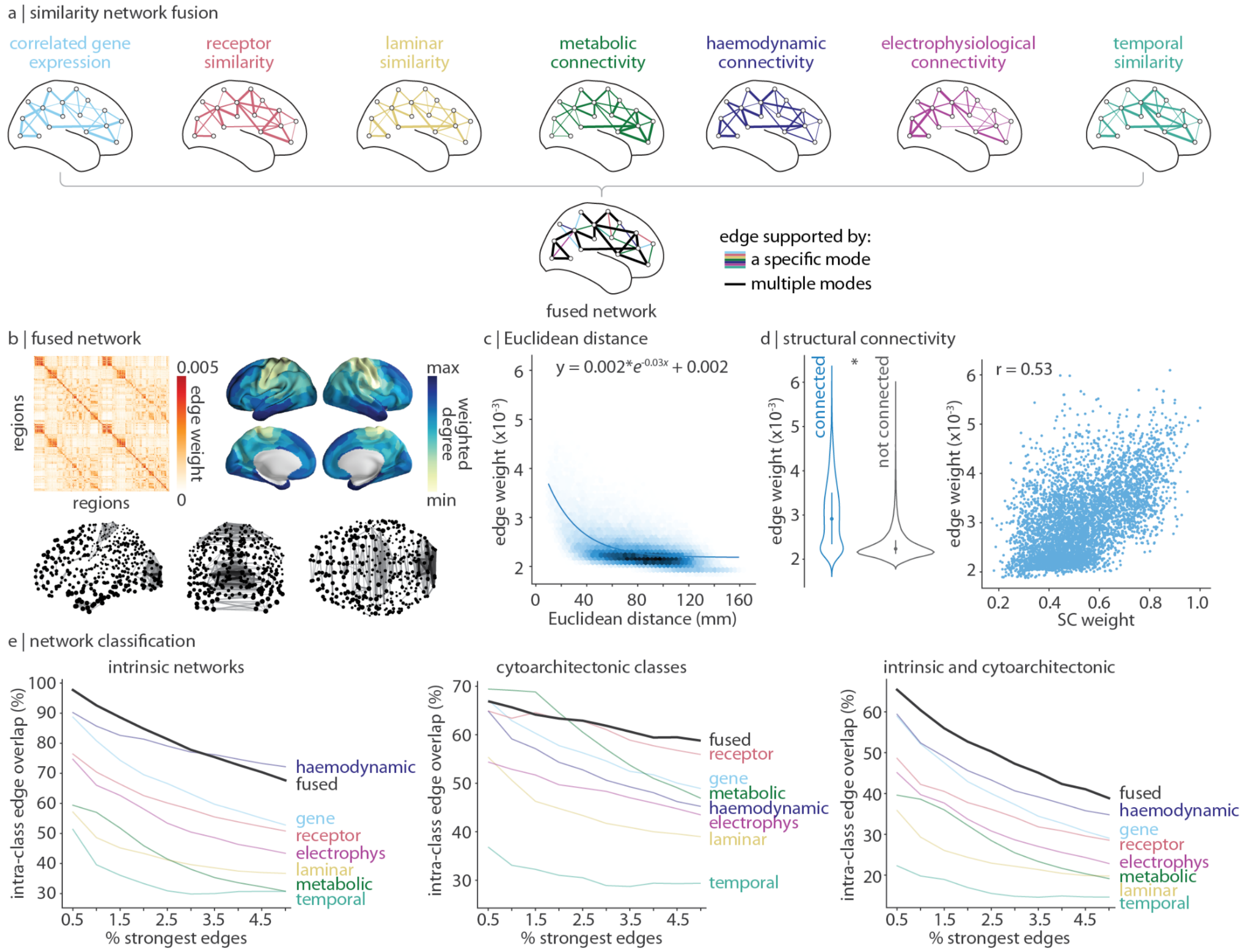

“Figure 6. Network fusion | Similarity network fusion was applied to all seven connectivity modes to construct a single integrated network (Wang et al 2014, Markello et al 2021). (a) Toy example of similarity network fusion (SNF). SNF iteratively combines the seven connectivity modes in a manner that gives more weight to edges between observations that are consistently high-strength across data types (black edges). (b) The fused network. We show the matrix-representation of the network (left), the top 0.5% strongest edges of the network (bottom), and the weighted degree of each brain region (right). Note that the edge weights in the fused network is a byproduct of the iterative multiplication and normalization steps (see *Methods* for details), and therefore can become very small. Greater edge magnitude represents greater similarity (no negative edges exist, by design). (c) Edge weight decreases exponentially with Euclidean distance. (d) Structurally connected edges have greater edge weight than edges without an underlying structural connection, against a degree and edge-length preserving null model (left (Betzel et al

> 2018)), and is correlated with structural connectivity (right). (e) For a varying threshold of strongest edges (0.5%--5% in 0.5% intervals), we calculate the proportion of edges that connect two regions within the same intrinsic network (left), cytoarchitectonic class (middle), and the union of intrinsic networks and cytoarchitectonic classes (right). "

We have updated the Results to include the presentation and interpretation of the new subplots ("Results" section, "Fusing connectivity modes" subsection, Paragraph #2):

> "The fused network's strongest edges exist between regions within somatomotor and visual cortex (Fig. 6b, bottom), likely reflecting the conserved molecular and dynamic composition of these phylogenetically older cortical regions. Meanwhile, cortical regions with the greatest weighted degree exist in anterior temporal and superior frontal cortex (Fig. 6b, right). The fused network exhibits non-random network organization including strong homotopic connectivity and a negative exponential relationships with distance (Fig. 6b left and Fig. 6c). In addition, structurally connected edges have significantly stronger edge weight than non-connected edges, against a degree- and edge-length preserving structural null (Fig. 6d). Finally, the fused network demonstrates a greater correlation between edge weight and weighted structural connectivity than any of the individual connectivity modes (r=0.53). This shows how combining inter-regional similarity across multiple scales can be used to better explain anatomical connectivity (Suarez et al 2020). This may be because regions that are similar across multiple scales are more likely to be connected, or because brain connectivity gives rise to shared biological features."

Minor comments:

10) I'd say that the definition of brain connectivity in the first line of the introduction refers more to the anatomical connectivity than other types of connectivity (e.g. functional connectivity).

This was deliberate - we start our Introduction with the more classical and expected definition of connectivity: anatomical connectivity. Later in paragraph 2, we introduce other forms of connectivity (e.g. functional connectivity and other correlation-based connectivity measures). The rationale was to establish the structural connectome as an oft-studied connectome that has resulted in many novel discoveries about the brain, but at the same time contrast this with the many other types of connectivity that can be derived and are worth studying.

11) In the sentence "An emerging representation of connectivity is feature similarity: if two brain regions exhibit similar biological features, we might expect them to be related to one another and engaged in common function" can you provide a reference for this definition?

We now provide five representative citations to articles, each showing how functionally similar regions have biologically similar features (neuroreceptors in Zilles et al 2015, gene expression in Anderson et al 2018, cytoarchitecture in Paquola et al 2019, and metabolism in Voigt et al 2022 and Horwitz et al 1984).

> "An emerging representation of connectivity is feature similarity: if two brain regions exhibit similar biological features, we might expect them to be related to one another and engaged in common function (Zilles et al 2015, Anderson et al 2018, Paquola et al 2019, Voigt et al 2022, Horwitz et al 1984)."

Anderson, K. M., Krienen, F. M., Choi, E. Y., Reinen, J. M., Yeo, B. T., & Holmes, A. J. (2018). Gene expression links functional networks across cortex and striatum. *Nature communications*, *9*(1), 1428.

Horwitz, B., Duara, R., & Rapoport, S. I. (1984). Intercorrelations of glucose metabolic rates between brain regions: application to healthy males in a state of reduced sensory input. *Journal of Cerebral Blood Flow & Metabolism*, *4*(4), 484-499.

Paquola, C., Vos De Wael, R., Wagstyl, K., Bethlehem, R. A., Hong, S. J., Seidlitz, J., ... & Bernhardt, B. C. (2019). Microstructural and functional gradients are increasingly dissociated in transmodal cortices. *PLoS biology*, *17*(5), e3000284.

Voigt, K., Liang, E. X., Misic, B., Ward, P. G., Egan, G. F., & Jamadar, S. D. (2023). Metabolic and functional connectivity provide unique and complementary insights into cognition-connectome relationships. *Cerebral Cortex*, *33*(4), 1476-1488.

Zilles, K., Bacha-Trams, M., Palomero-Gallagher, N., Amunts, K., & Friederici, A. D. (2015). Common molecular basis of the sentence comprehension network revealed by neurotransmitter receptor fingerprints. *Cortex*, *63*, 79-89.

12) While the meaning of brain structure is straightforward, the one of "brain geometry" has been used and misused in the literature, so I think the readers would benefit from an accurate definition of this concept.

Agreed. By "geometry" we mean the brain's spatial embedding in 3D Euclidean space, but the only geometric feature we study is distance (between brain regions). We therefore opted to use "distance" rather than geometry in most places, and to explicitly label "distance" and "brain geometry" early in the Results.

("Results" section, "Common organizational patterns of connectivity modes" subsection, Paragraph #3):

> "Altogether, we find that connectivity modes demonstrate commonalities that respect distance, neuroanatomy, and anatomical connectivity, regardless of imaging modality or biological mechanism."

("Results" section, "Structural and geometric features of connectivity modes" subsection, Paragraph #1):

> "We focus on two metrics to classify edges between cortical regions: distance (brain geometry) and structural connectivity (brain structure)."

13) Is it 79800 the number of possible edges, given the chosen brain parcellation? If so, please specify it.

Yes - we have now clarified this in the text:

("Results" section, "Structural and geometric features of connectivity modes" subsection, Paragraph #1):

> "To directly compare edge weights across connectivity modes, we converted edge weights to ranks, such that the smallest (i.e. most negative) edge is ranked 1 and the

strongest (i.e. most positive) edge is ranked 79,800 (equal to the number of edges in each network, under the 400-region Schaefer parcellation)."

Figure 2 caption:

"Edges are binned into 50 equally-sized bins of increasing Euclidean distance (79,800 edges total under the 400-region Schaefer parcellation, 1,596 edges per bin). For each connectivity mode, the median edge rank is plotted within each bin."

14) Some sections are named with the type of measure extracted or analysis performed, e.g., "Cross-modal hubs" and "Fusing connectivity modes", others with the message that the authors want to convey with that section, e.g. "Connectivity modes shape disease vulnerability". I think that a unique approach to define each section would be easier to follow, and I find the latter more useful than the former.

In an effort to keep subsection titles short, and because some subsections have multiple main findings, we have modified the subsection titles such that they are all named based on the type of measure extracted or analysis performed (i.e. the Reviewer's option 1). Therefore we only changed the subheading for "Connectivity modes shape disease vulnerability" (now "Connectivity modes and disease-specific abnormal cortical thickness").

15) The last sentence of section "Connectivity modes shape disease vulnerability" is: "This integrative analysis makes it possible to hone in on the imaging modalities and biological mechanisms that might most reflect cortical pathology; in this case, bringing to light the relevance of molecular rather than dynamic modes in psychiatric disorders." It's not unexpected or a new concept that the molecular layer of the brain is relevant for the study of psychiatric disorders. I would change "bringing to light" with "confirming".

We agree with the Reviewer and have revised the manuscript to better contextualize the results with previous studies, as well as clarify the novelty of the present study. We have removed the phrase "bringing to light" in the restructuring of the sentence, also in response to Reviewer #1 Comment #3.

("Results" section, "Connectivity modes and disease-specific abnormal cortical thickness" subsection, Paragraph #1):

"Pioneering studies in post-mortem tissue gave rise to the theory that the physicochemical composition of neurons at local brain regions results in a selective vulnerability to brain disease (Klatzo 2003). Other classical studies have shown that disease propagation in the cerebral cortex is related to microscale features such as myelination (Braak & Braak 1996). These propagation patterns have been successfully modeled at the level of the whole-cortex using the structural connectome, and often perform better when informed by local biological features such as the expression of a specific gene (Henderson et al 2019, Shafiei et al 2023). Recent findings build on this notion and posit that the course and expression of multiple brain diseases on the structural connectome is mediated by multiple forms of molecular vulnerability rather than a single molecular perturbation (Warren et al 2013, Hansen et al 2022). We therefore tested whether disease propagation patterns derived from the connectivity modes could predict abnormal cortical thickness patterns for thirteen different neurological, psychiatric, and neurodevelopmental diseases and disorders from the ENIGMA consortium

(N=21,000 patients, N=26,000 controls) (Thompson 2020, Lariviere et al 2021, Hansen et al 2022). The disease-specific abnormal cortical thickness patterns are regional z-scored case-versus-control effect sizes, representing deviation from normative cortical thickness. We refer to these regional values as “abnormal cortical thickness” or simply “abnormality”.”

(“Results” section, “Connectivity modes and disease-specific abnormal cortical thickness” subsection, Paragraph #3):

> “This integrative analysis makes it possible to hone in on the imaging modalities and biological mechanisms that might most reflect cortical pathology in a disease-general manner. Furthermore, it demonstrates the value in employing feature similarity as a network rather than limiting network models to the structural connectome.”

16) In the electrophysiological connectivity section in the methods, I'd find more intuitive to have the description of the processing steps before the part related to the estimation of the connectivity matrix.

Agreed. We have reordered the subsection such that the preprocessing steps are described before the estimation of the connectivity matrix (“Methods” section, “Connectivity modes” subsection, “Electrophysiological connectivity” subsubsection):

> “Electrophysiological connectivity was measured using magnetoencephalography (MEG) recordings, which tracks the magnetic field produced by neural currents. Resting state MEG data was acquired for n=33 unrelated healthy young adults (age range 22--35 years) from the Human Connectome Project (S900 release (van Essen et al 2013)). The data includes resting state scans of approximately 6 minutes long and noise recording for all participants. MEG anatomical data and 3T structural MRI of all participants were also obtained for MEG pre-processing.
>
> The present MEG data was first processed and used by Shafiei et al 2022. Resting state MEG data was preprocessed using the open-source software, Brainstorm (https://neuroimage.usc.edu/brainstorm; (Tadel et al 2011)), following the online tutorial for the HCP dataset (https://neuroimage.usc.edu/brainstorm/Tutorials/HCP-MEG). MEG recordings were registered to individual structural MRI images before applying the following preprocessing steps. First, notch filters were applied at 60, 120, 180, 240, and 300 Hz, followed by a high-pass filter at 0.3 Hz to remove slow-wave and DC-offset artifacts. Next, bad channels from artifacts (including heartbeats, eye blinks, saccades, muscle movements, and noisy segments) were removed using Signal-Space Projections (SSP).
>
> Pre-processed sensor-level data was used to construct a source estimation on HCP's fsLR4k cortex surface for each participant. Head models were computed using overlapping spheres and data and noise covariance matrices were estimated from resting state MEG and noise recordings. Linearly constrained minimum variance (LCMV) beamformers was used to obtain the source activity for each participant. Data covariance regularization was performed and the estimated source variance was normalized by the noise covariance matrix to reduce the effect of variable source depth. All eigenvalues smaller than the median eigenvalue of the data covariance matrix were replaced by the

median. This helps avoid instability of data covariance inversion caused by the smallest eigenvalues and regularizes the data covariance matrix. Source orientations were constrained to be normal to the cortical surface at each of the 8,000 vertex locations on the cortical surface, then parcellated according to the Schaefer-400 atlas (Schaefer et al 2018).

After preprocessing and parcellating the data, amplitude envelope correlations were performed between time-series at each pair of brain regions, for six canonical frequency bands separately (delta (2--4 Hz), theta (5--7 Hz), alpha (8--12 Hz), beta (15--29 Hz), low gamma (30--59 Hz), and high gamma (60--90 Hz)) (Bruns et al 2000). Amplitude envelope correlation is applied instead of directly correlating the time-series because of the high sampling rate (2034.5 Hz) of the MEG recordings. An orthogonalization process was applied to correct for the spatial leakage effect by removing all shared zero-lag signals (Colclough et al 2016). The composite electrophysiological connectivity matrix is the first principal component of all six connectivity matrices (vectorized upper triangle), and closely resembles alpha connectivity (Figure S8). Finally, the matrix underwent Fisher's r-to-z transform."
